# High Fe-Loading Single-Atom Catalyst Boosts ROS Production by Density Effect for Efficient Antibacterial Therapy

**DOI:** 10.1007/s40820-024-01522-1

**Published:** 2024-10-04

**Authors:** Si Chen, Fang Huang, Lijie Mao, Zhimin Zhang, Han Lin, Qixin Yan, Xiangyu Lu, Jianlin Shi

**Affiliations:** 1 Department of Cardiology, Shanghai Tenth People’s Hospital, School of Medicine, Tongji University, Shanghai, 200092 People’s Republic of China; 2 Shanghai Institute of Ceramics, Chinese Academy of Sciences, Shanghai, 200050 People’s Republic of China; 3 Shanghai Frontiers Science Center of Nanocatalytic Medicine, School of Medicine, Tongji University, Shanghai, 200092 People’s Republic of China

**Keywords:** Nanocatalytic medicine, Single-atom catalysts, Reactive oxygen species (ROS), High metal loading, Oxidase catalysis

## Abstract

The current single-atom catalysts (SACs) for medicine still suffer from the limited active site density. Here, we develop a synthetic method capable of increasing both the metal loading and mass-specific activity of SACs by exchanging zinc with iron. The constructed iron SACs (h^3^-FNC) with a high metal loading of 6.27 wt% and an optimized adjacent Fe distance of ~ 4 Å exhibit excellent oxidase-like catalytic performance without significant activity decay after being stored for six months and promising antibacterial effects. Attractively, a “density effect” has been found at a high-enough metal doping amount, at which individual active sites become close enough to interact with each other and alter the electronic structure, resulting in significantly boosted intrinsic activity of single-atomic iron sites in h^3^-FNCs by 2.3 times compared to low- and medium-loading SACs. Consequently, the overall catalytic activity of h^3^-FNC is highly improved, with mass activity and metal mass-specific activity that are, respectively, 66 and 315 times higher than those of commercial Pt/C. In addition, h^3^-FNCs demonstrate efficiently enhanced capability in catalyzing oxygen reduction into superoxide anion (O_2_·^−^) and glutathione (GSH) depletion. Both in vitro and in vivo assays demonstrate the superior antibacterial efficacy of h^3^-FNCs in promoting wound healing. This work presents an intriguing activity-enhancement effect in catalysts and exhibits impressive therapeutic efficacy in combating bacterial infections.

**Supplementary Information:**

The online version contains supplementary material available at 10.1007/s40820-024-01522-1.

## Highlights

- Fe single-atom catalysts (h^3^-FNCs) with high loading, high catalytic activity and high stability were synthesized via a method capable of increasing both the metal loading and mass-specific activity by exchanging zinc with iron.
- The “density effect,” derived from the sufficiently high density of active sites, has been discovered for the first time, leading to a significant alteration in the intrinsic activity of single-atom metal sites.
- The superior oxidase-like catalytic performance of h^3^-FNCs ensures highly effective bacterial eradication.

**Supplementary Information:**

The online version contains supplementary material available at 10.1007/s40820-024-01522-1.

## Graphical abstract

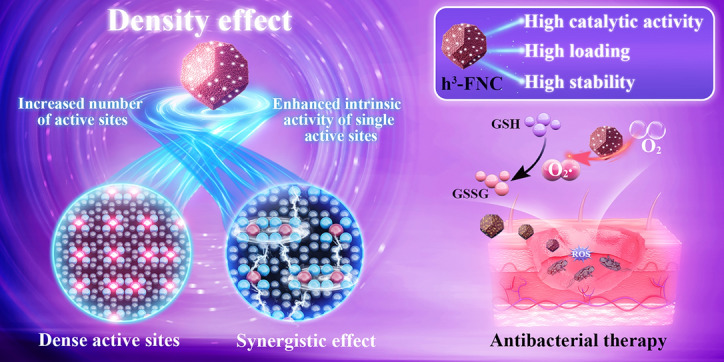

## Introduction

While catalytic medicine has gained significant attention in recent years, the complexity of the chemical components and structures of most nanomedicines, coupled with the difficulty in identifying active sites, establishes substantial hurdles in the way of their clinical translation [1–4]. Therefore, a primary goal of catalyst design for medical purposes should be to achieve optimal therapeutic effects by using as simple as possible material composition. In this context, single-atom catalysts (SACs), featuring designable active centers, ultrahigh atom utilization, and simple compositions and structures, hold great potential in the medical field [5–7]. However, as emerging medical materials, SACs are still in the early stages of exploration, and their catalytic activity needs to be further optimized to achieve ideal therapeutic effects [8, 9]. Generally, two strategies can be employed to optimize the performance of SACs: (1) increasing the density of active metal atoms and (2) elevating the intrinsic activity of single-atom sites. Generally, the two strategies mentioned above cannot be achieved at the same time. Several studies have attempted to improve individual intrinsic activity by modulating the local coordination environments of the active sites or introducing different metal atoms for synergistic catalysis [10, 11], but these efforts have only achieved limited success due to the over-low quantities of active sites. Alternatively, current SACs applied in the medical field typically exhibit relatively low metal loadings, often approximately 2%, or even below 0.2% primarily due to the undesired formation of metallic clusters or even nanoparticles at enhanced loadings (Table S1) [12]. Therefore, it is of great significance to develop a strategy to elevate the metal loading of SACs for density modulation while enhancing the intrinsic activity of single-atom sites.

Microorganisms, ubiquitous in the human living environment, can invade the human body in numerous ways, triggering a series of diseases. Consequently, bacterial infections are among the most common diseases in humans [13]. Despite the existence of a wide array of antimicrobial therapeutics, the practical application of these therapies is significantly undermined by a number of factors such as bacterial resistance, low antibacterial efficiency, complicated treatment processes and high cost [14, 15]. In addition, antimicrobial drugs, such as phage-derived peptides, antibodies, and vaccines, usually demand specific preservation conditions and have limited shelf lives [16]. Recently, nanocatalytic medicine has gained extensive attention due to its broad-spectrum antibacterial activity, achieved by catalyzing the production of reactive oxygen species (ROS) that induce oxidative stress, effectively annihilating bacteria while reducing the likelihood of bacterial resistance [17–20]. However, these studies rely mostly on hydrogen peroxide as the substrate for Fenton reactions or peroxidase-like reactions to produce ROS; resultantly, their efficacies are greatly diminished by the insufficient presence of hydrogen peroxide in the microenvironment of bacterial infections [21]. Alternatively, catalysts with oxidase-like activity are much more effective and desirable than other candidates for antibacterial application owing to the sufficient supply of O_2_ without the need for any external stimulation.

**Fig. 1:**
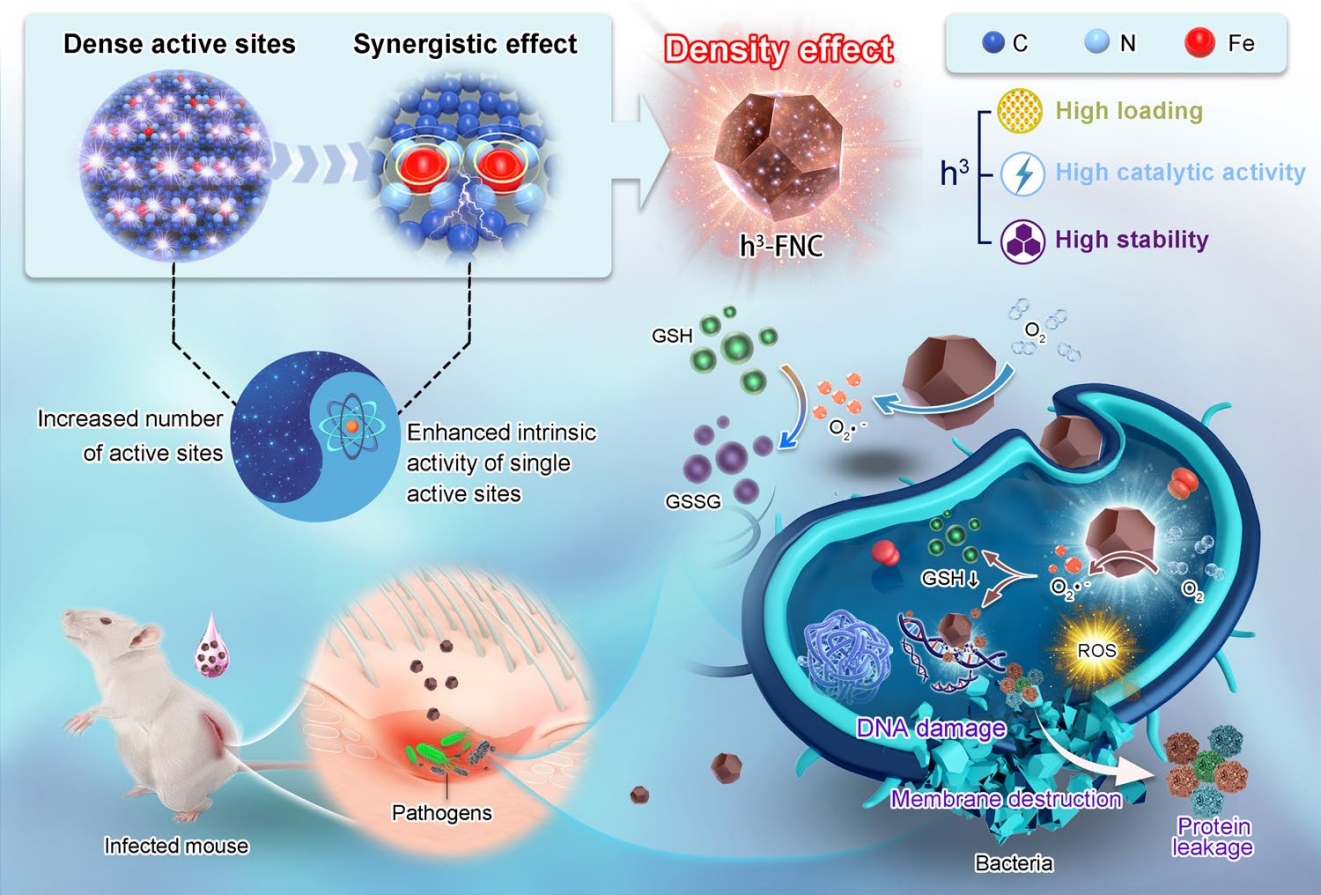
Schematic illustration of the antibacterial applications of h^3^-FNCs with density effect. Single Fe atoms distribute densely in the high-loading single-atom catalysts (h^3^-FNCs), leading to neighboring Fe–N_4_ moieties being close enough to each other, and resultantly, the largely enhanced intrinsic oxidase-like activity of single active sites from the interaction between the adjacent metallic sites. Such a novel activity-alteration phenomenon is termed herein as “density effect.” The present h^3^-FNCs can efficiently catalyze ROS production and GSH depletion to cause membrane destruction, DNA damage, and protein leakage in the infection region, leading to the effective eradication of *S. aureus* and *P. aeruginosa* both in vitro and in vivo

Here, we have developed a novel synthetic method to construct a “triple-high” iron medical single-atom catalyst, designated as h^3^-FNC, by harnessing the abundant atomical Zn sites in ZIF-8 and strategically decoupling the formation of isolated Fe–N sites from pyrolysis. The resultant h^3^-FNCs, characterized by an especially high Fe loading (6.27 wt%), high oxidase-like activity and high stability, demonstrate improved therapeutic efficacies with a 97.8% wound healing rate and excellent biosafety (Fig. 1). And the features are as follows: (1) The metal mass-specific activity of h^3^-FNCs significantly surpasses those of the low- and medium-loading single-atom catalysts. (2) At a high-enough density of active sites, the distance between neighboring metal sites decreases to a critical value (~ 4 Å in this work), leading to the notable electronic interaction between the adjacent metal atom sites which largely enhances the intrinsic activity of single-atomic active sites for enzyme-mimic catalysis. This phenomenon wherein variations in the density of active sites influence the activity of individual catalytic sites is refferred to as “density effect,” which is derived from the interactions among adjacent sites at the elevated density of single metal active sites. (3) The majority of active sites are distributed on the surface of h^3^-FNCs and can be fully utilized in catalysis. As a result, h^3^-FNCs exhibit an especially high oxidase-like catalytic performance, and their mass activity and metal mass-specific activity are, respectively, 66 and 315 times higher than those of commercial Pt/C. In addition, h^3^-FNCs can efficiently catalyze oxygen (O_2_) reduction into superoxide anion (O_2_·^−^) and glutathione (GSH) depletion, favoring damaging bacterial membranes. Density functional theory (DFT) calculations further reveal the fundamental mechanism of the density effect in h^3^-FNCs for the oxidase-like performance. Besides, the catalytic performance of h^3^-FNCs shows almost no decay after storage for six months, ensuring convenient storage, transportation and long-lasting therapeutic effects.

## Experimental Section

### Materials

2-Methylimidazole, dicyandiamide, 3,3′,5,5′-tetramethylbenzidine dihydrochloride hydrate (TMB), dihydroethidium (DHE), 2,7-dichlorofluorescein diacetate (DCFH-DA) were purchased from Sigma-Aldrich Co., Ltd. Zinc nitrate hexahydrate (Zn(NO_3_)_2_·6H_2_O) was obtained from Alfa Aesar. Concentrated sulfuric acid (H_2_SO_4_, 95%) and hydrogen peroxide aqueous solution (H_2_O_2_, 30 wt%) were purchased from Shanghai Chemical Reagents, China. Ferrous chloride tetrahydrate (FeCl_2_·4H_2_O) was purchased from Thermo Fisher Scientific Inc. Commercial 60% Pt/C and 30% Pt/C (Johnson Matthey) were purchased from Shanghai Hephas Energy Co., Ltd. 5,5-dimethyl-1-pyrroline N-oxide (DMPO) was purchased from Shanghai Dojindo Co., Ltd. Reduced glutathione (GSH) were purchased from Aladdin Chemical Reagent Co., Ltd. Cellular glutathione peroxidase assay kit with DTNB was purchased from Beyotime Biotechnology Co., Ltd. SYTO-9/PI live/dead bacterial double stain kit was purchased from shanghai Maokang biotechnology Co., Ltd. Human skin fibroblasts (HSF) were obtained from Jennio Biotech Co., Ltd (China). Staphylococcus aureus (ATCC6538) and Pseudomonas aeruginosa (ATCC9027) were ordered from Guangdong Microbial Culture Collection Center (Guangzhou, China). All chemicals were used without further purification.

### Characterization

Scanning electron microscopy (SEM) images were operated on a ZEISS GeminiSEM 300 microscope with an accelerating voltage of 15 kV. X-ray diffraction (XRD) patterns were obtained using a Bruker D8 Advance X-ray powder diffractometer. Transmission electron microscopy (TEM) images were recorded on a JEOL JEM 2100F. Aberration-corrected high-angle annular dark-field scanning transmission electron microscopy (AC HAADF-STEM), high-resolution STEM (HR-STEM), and corresponding energy-dispersive X-ray spectroscopy (EDXS) mapping were recorded on an EM-ARM300F with spherical aberration correction operated at 300 kV. The concentration of Fe was determined by inductively coupled plasma optical emission spectrometry (ICP-OES, Agilent 700 Series, Agilent Technologies). The N_2_ adsorption/desorption isotherm and pore structures were measured by a Micromeritics Tristar II 3020 instrument. Pore volumes were determined by the non-local density functional theory method using Microactive for Tristar II software. FTIR spectra were measured using a Thermo Scientific Nicolet iS20 spectrometer with KBr powder as the background correction. The Raman spectra were obtained on the Horiba LabRAM HR Evolution spectrometer at room temperature. The X-ray photoelectron spectroscopy (XPS) of the N 1*s* spectra were acquired using an ESCAlab250 (Thermal Fischer). The Mössbauer measurements were performed at room temperature using a conventional spectrometer (Germany, Wissel MS-500) in transmission geometry with constant acceleration mode. Electron paramagnetic resonance (EPR) experiments were conducted on a Bruker EMXnano spectrophotometer. Fluorescence measurements were performed on a Hitachi F-4600 fluorescence spectrophotometer. Flow cytometry analysis was performed on a Beckman Coulter CytoFLEX cytometer. The relevant software required for DFT calculations is copyrighted by Hangzhou Yanqu Information Technology Co., Ltd.

### Preparation of NCs and FNCs

Zinc nitrate hexahydrate (5.95 g) and dimethylimidazole (6.57 g) were added to methanol solution (300 mL) and stirred at room temperature for 6 h to synthesize ZIF-8. Then, Zn–NCs were obtained by pyrolyzing ZIF-8 at 900 °C for 1 h (heating rate of 3 K min^−1^) in N_2_ atmosphere, then refluxed in H_2_SO_4_ solution (2 M) for 16 h to exfoliate the Zn to obtain NCs, which contained abundant vacancies. NCs (0.5 g) were dispersed in methanol (200 mL) containing the same mass of FeCl_2_·4H_2_O and refluxed for 16 h with continuous stirring to produce Fe–NCs. After fully washing, Fe–NCs were immersed in H_2_SO_4_ solution (0.5 M) for 12 h to fully remove the physisorbed Fe ions and thoroughly washed with water. Afterward, dicyandiamide was mixed with the dried Fe–NCs in a 1:2 mass ratio and ground for 15 min. The mixture was activated at 900 °C for 1 h in a gas mix of N_2_ containing 5% H_2_ (same heating rate as above) to obtain h^3^-FNC catalysts. Samples of m-FNC and l-FNC were prepared by the same procedure except that the mass ratio of FeCl_2_·4H_2_O to NC was 1:2 and 1:6, respectively, during sample synthesis.

## Results and Discussion

### Design Principle and Structural Characterizations

**Fig. 2:**
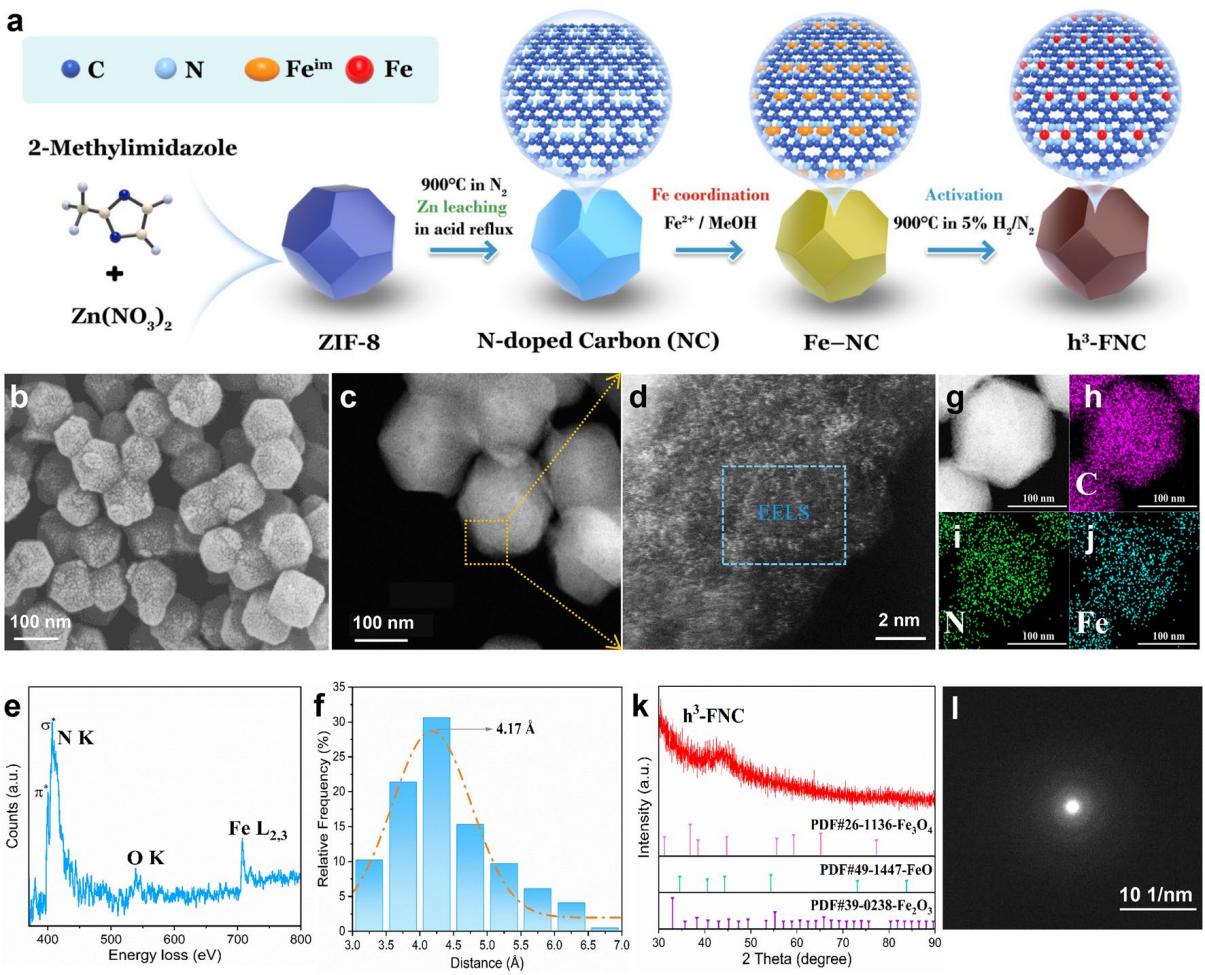
Morphological and structural characterization. **a** Synthetic method for h^3^-FNCs with high Fe loading. Initially, ZIF-8 was synthesized using zinc nitrate hexahydrate and dimethylimidazole as the starting precursors, followed by pyrolyzing at 900 °C and refluxing in acidic medium to leach out Zn, obtaining a nitrogen-doped carbon (NC) matrix with abundant vacancies. After coordination with Fe ions, Fe–NC was activated at 900 °C in the presence of dicyandiamide to obtain h^3^-FNC. (Fe^im^: the intermediate state of Fe). **b** SEM and **c** TEM images of h^3^-FNCs. **d** Atomic-resolution AC HAADF-STEM micrograph of the area marked in **c**, highlighting single Fe atoms as bright dots. **e** EELS of the blue-framed area in **d**. **f** Kernel method of frequency statistic and corresponding Gauss fitting of diatomic distances acquired from the Fe atoms in **d**. **g** HAADF-STEM image and corresponding EDS mappings of h^3^-FNCs: **h** C, **i** N, and **j** Fe. **k** XRD pattern and **l** SAED pattern of h^3^-FNCs

The synthesis process of h^3^-FNCs is depicted in Fig. 2a. Initially, a Zn-based metal–organic framework (ZIF-8) was prepared by mixing 2-methylimidazole and zinc nitrate hexahydrate with stirring. Subsequent pyrolysis yielded a nitrogen-doped carbon substrate enriched with Zn active sites (Zn–NC). These Zn sites were then leached by H_2_SO_4_ etching, forming a vacancy-enriched nitrogen-doped carbon matrix (NC). Fe atoms were then introduced into the matrix, producing Fe atom-densely dispersed catalyst on the NC surface (Fe–NC). The final h^3^-FNC was obtained by the activation of Fe–NC under N_2_ atmosphere containing 5% H_2_.

The morphological and structural properties of h^3^-FNC were investigated using a series of techniques. SEM (Fig. 2b) and TEM images (Fig. S1) reveal the polyhedral morphology of h^3^-FNCs consistent with that of ZIF-8 and uniform dispersion (Fig. S2). AC HAADF-STEM image of h^3^-FNCs identify densely populated but isolated Fe atoms (Fig. 2c, d). Fe amount was further determined to be 6.27 wt% by ICP-OES. This high loading is a prerequisite for the density effect. Electron energy loss spectroscopy (EELS) analysis (Fig. 2e) exhibits a clear Fe signal originating from the isolated Fe atoms within the blue-squared region in Fig. 2d. Meanwhile, statistical analysis of the representative single atoms in Fig. 2d (also Fig. S3) reveals that the most probable distance between adjacent Fe atoms ranges from 3.5 to 5.5 Å, and the prevalence is maximized at approximately 4.17 Å according to Gaussian fitting (Fig. 2f). From the result of inter-site distances, a high proportion of Fe atoms is distributed in close proximity in h^3^-FNCs.

In addition, the HAADF-STEM images and the corresponding energy-dispersive X-ray spectroscopy (EDS) mappings demonstrate the homogeneous distribution of C, N, and Fe elements in the carbon matrix, with a rather strong signal of Fe reflecting the high Fe loading (Fig. 2g–j). The XRD (Fig. 2k) and selected-area electron diffraction (SAED) patterns (Fig. 2l) indicate the poor crystallinity of h^3^-FNC. Moreover, the absences of characteristic peaks in the XRD pattern and diffraction spots in the SAED pattern of iron-based crystalline phases suggests that iron-containing clusters or particles are not present.

### Atomic Structure Analysis

**Fig. 3:**
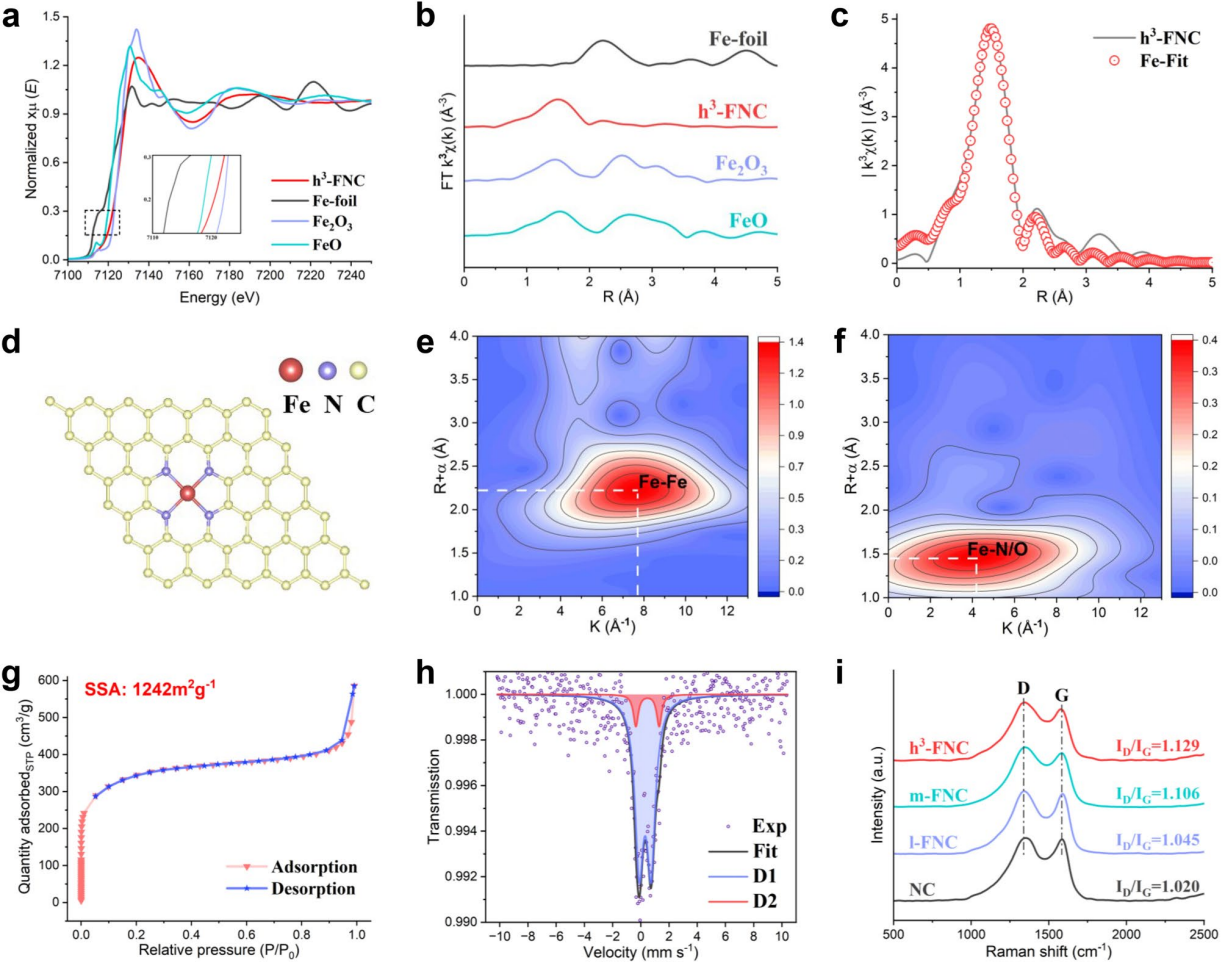
Fine structure analysis.** a** Fe K-edge XANES spectra. **b** Fourier transforms of k^2^-weighted χ(k)-function of EXAFS spectra. **c** Fe K-edge EXAFS fitting results of h^3^-FNCs in R space without a phase-shift correction. **d** Proposed architecture of Fe single sites in h^3^-FNCs. Wavelet transforms of the k^2^-weighted Fe K-edge EXAFS signals of** e** Fe foil and **f** h^3^-FNCs. **g** N_2_ adsorption and desorption isotherms of h^3^-FNCs. **h** ^57^Fe Mӧssbauer spectra of h^3^-FNCs at ambient temperature. **i** Raman spectra of the catalysts

A series of fine structure tests, such as X-ray absorption spectroscopy (XAS), Mössbauer spectroscopy, and XPS, were performed to analyze the chemical structure and coordination environment of Fe atoms. Figure 3a exhibits the Fe *K*-edge X-ray absorption near-edge structure (XANES) profiles of h^3^-FNCs using Fe foil, FeO and Fe_2_O_3_ as the references. The valence state of Fe in h^3^-FNCs is in between  + 2 and  + 3 judged from the positions of its absorption and edge-front peaks. The Fourier transform of the extended X-ray absorption fine structure (EXAFS) spectrum clearly shows the absence of peaks belonging to Fe–Fe bonds at 2.13 Å, indicating that the Fe atoms are isolated from each other in the h^3^-FNC, most probably forming atomically dispersed Fe–N sites (Fig. 3b). Based on the structural parameters obtained by XAFS fitting results (Figs. 3c, S4 and Table S2), the coordination number of the Fe–N first coordination shell is 4, implying that the dominant Fe–N structure is Fe–N_4_ (Fig. 3d). This Fe–N_4_ coordination strongly resembles the iron porphyrin core in natural oxidases, such as cytochrome P450 [22] and nitric oxide synthase [23], which are highly active in catalyzing redox reactions. Notably, the Fe atoms are axially coordinated to an oxygen atom in the first coordination shell, in addition to the four surrounding nitrogen atoms, as this single-atom Fe is so reactive and therefore highly susceptible to oxygen adsorption [24]. In addition, we further investigated the wavelet transform (WT) analysis to verify the backscattered atoms around the active site. The WT plot of Fe foil (Fig. 3e) shows the intensity maxima corresponding to the Fe–Fe bond, which is missing in the WT plot of h^3^-FNCs (Fig. 3f), further confirming the absence of metal-based nanoparticles in h^3^-FNCs. The first intensity maximum of h^3^-FNCs at 1.45 Å and a corresponding *k* value of ~ 4.2 Å^−1^ can be attributed to Fe–N and Fe–O bonds. WT analysis corroborates the presence of Fe single-atom sites in h^3^-FNCs in accordance with the EXAFS fitting parameters.

In most single-atom catalysts, metal atoms tend to homogeneously distribute through the matrix, rather than be located on the surface, leading to the most active sites being embedded in the interior of the matrix. Consequently, in this case, the amount of active site accessible to reactants will scale up with the increase of the specific surface area (SSA) of the catalyst. Therefore, the N_2_ sorption isotherms were obtained to evaluate the SSA of h^3^-FNCs. As shown in Fig. 3g, h^3^-FNCs exhibit type I isotherms without significant hysteresis, indicating the predominant microporous structures. Employing the Brunauer–Emmett–Teller method, the SSA of h^3^-FNCs was calculated to be 1242 m^2^ g^–1^, much higher than most reported Fe–N–C catalysts in the range of 600–800 m^2^ g^–1^ [25], enabling h^3^-FNCs to expose most active Fe atomic sites on the outer and micropore surfaces, thanks to the high SSA and substantial microporous volume (Fig. S5). Furthermore, Mössbauer spectroscopy was adopted to determine the location, chemical environment and spin state of Fe in h^3^-FNCs [26]. The resulting ^57^Fe Mössbauer spectrum of h^3^-FNCs is deconvoluted into two independent doublets labeled as D1 and D2 according to isomer shifts (IS) and nuclear quadrupole splitting (QS) (Fig. 3h and Table S3). No sextets or singlets are present in the room-temperature spectrum, indicating that there are no super-paramagnetic non-nanometric iron side phases such as metallic iron or iron carbide in h^3^-FNCs [27]. This result indicates that our synthetic approach is much more desirable than conventional pyrolytic Fe–N–C syntheses, in which particles of metallic iron or iron carbide may exist and are hard to remove [12]. Although both D1 and D2 have similar IS with each other, their QS values significantly differ from each other, where D1 exhibits a QS of 0.84 mm S^−1^, in contrast to D2 of 1.65 mm S^−1^ in QS value. More specifically, D1 can be identified as a ferric high-spin Fe–N site which is located on the carbon surface and with oxygen adsorbate(s), while the minor D2 signal corresponds to a ferrous low- or medium-spin Fe–N site which might be buried in the bulk, or on the carbon surface but with weaker oxygen binding [28, 29]. The D1/D2 value was calculated to be 8.9, indicating that the vast majority of iron atoms are located on the surface of h^3^-FNC accessible to the reactant molecules, which is an important factor for the especially high catalytic activity of h^3^-FNC as presented in the following section.

To investigate the relationship between metal content and catalytic activity in SACs, two other catalysts with different iron loadings were synthesized by the same method: a low-loading (0.71 wt%) single-atom catalyst (l-FNC) and a medium-loading (4.14 wt%) single-atom catalyst (m-FNC). These FNCs were structurally analyzed by Fourier transform infrared (FTIR) spectroscopy, Raman spectroscopy and X-ray photoelectron spectroscopy (XPS). The FTIR spectra exhibit the characteristic absorption peaks for C–N stretching vibration, C = C stretching vibration and O–H stretching vibration at 1394, 1632, and 3425 cm^−1^, respectively (Fig. S6). This indicates that the introduction of Fe single atoms has resulted in accumulated hydroxyl groups on the surface of the particles, beneficial for their biomedical catalytic applications [30]. The D (disorder) and G (graphite) peaks appearing in Fig. 3i reflect the carbon structure and defect density within the sample [31]. The *I*_D_/*I*_G_ values of h^3^-FNCs, m-FNCs, l-FNCs, and NC catalysts are 1.129, 1.106, 1.045, and 1.020, respectively, indicating that h^3^-FNCs have the lowest degree of graphitization, the highest defect concentration, and resultantly the highest surface energy, which greatly facilitates chemical reaction kinetics [32]. The type of nitrogen atoms in h^3^-FNCs was investigated by XPS. The N 1*s* XPS spectrum was deconvoluted into five peaks corresponding to pyridine N (398.2 eV), metal–N (399.5 eV), pyrrole N (400.5 eV), graphite N (401.8 eV) and oxidized N (403.8 eV) (Fig. S7), showing the predominance of pyridinic, pyrrolic, and metal-ligated N sites [33].

### Oxidase-Like Activity Assays

**Fig. 4:**
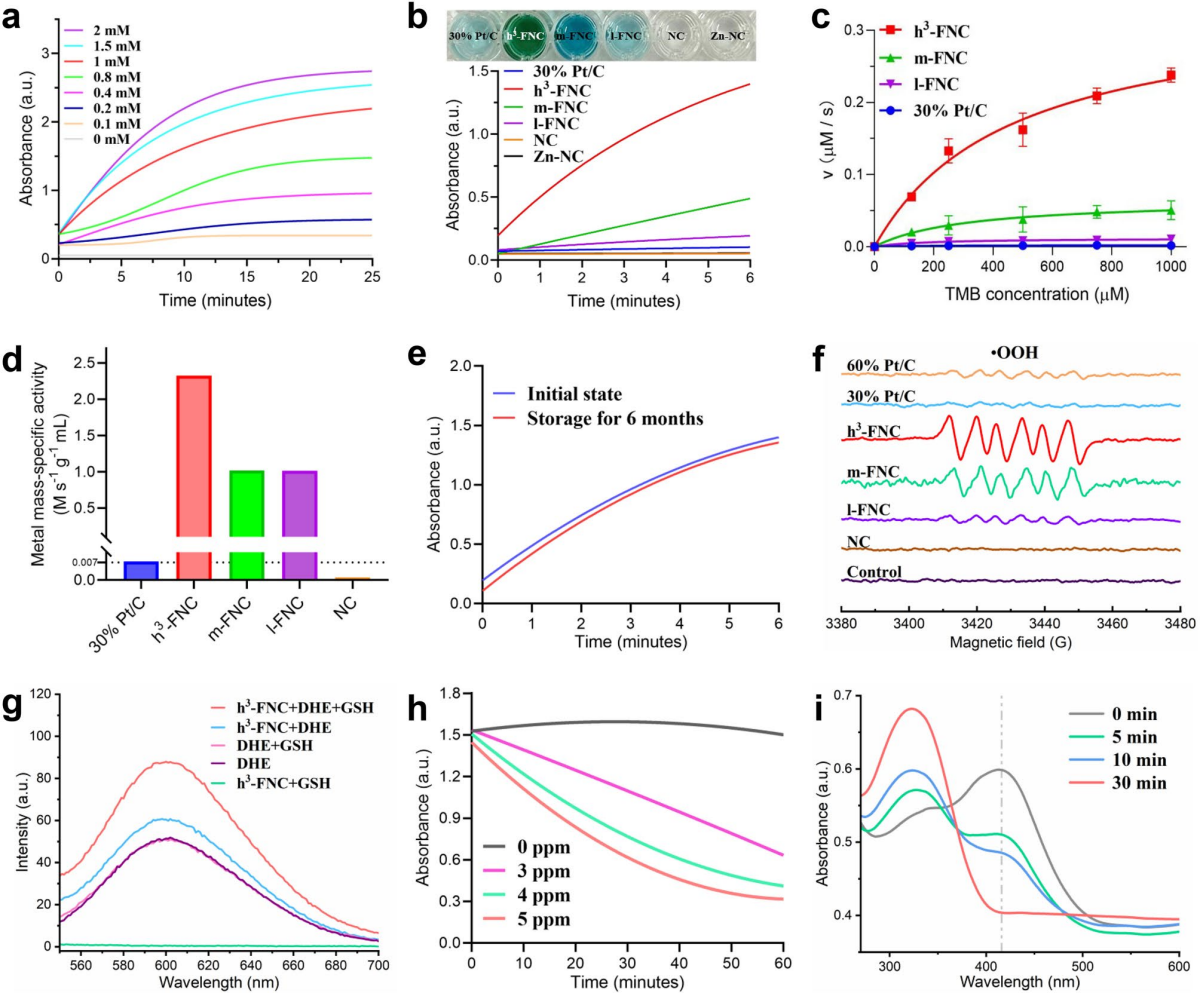
Catalytic performance of the catalysts. **a** Time-dependent absorbance changes of TMB of varied concentrations in the presence of h^3^-FNCs (1 ppm) in deionized water. **b** Absorption intensity of TMB (1 mM) in the presence of different catalysts (1 ppm) in deionized water. Inset: the corresponding digital photos of each group. **c** Velocity (*v*) of the chromogenic reaction of TMB in the presence of different materials in deionized water. **d** Metal mass-specific activity of different catalysts calculated from the reaction rates in **c**. **e** Long-term catalytic stability assessment of h^3^-FNCs as monitored by detecting the oxidation of TMB. **f** EPR spectra of DMPO/·OOH. **g** Fluorescence spectra of DHE with h^3^-FNC, with or without the addition of GSH. **h** GSH consumption by different concentrations of h^3^-FNCs using DTNB as the indicator. **i** GSH depletion performance of h^3^-FNCs (10 ppm) at different time points using DTNB as an indicator by UV–vis absorption. ppm: μg mL.^−1^

With its iron site similar to that of Fe oxidase [22], h^3^-FNCs will potentially exhibit enhanced oxidase-like activity. Therefore, we used the oxidation of 3,3′,5,5′-tetramethylbenzidine (TMB) as a model catalytic reaction to investigate the catalytic activity of h^3^-FNCs at room temperature in catalyzing the generation of O_2_·^−^ from ambient oxygen. As shown in Figs. 4a, b and S8a–d, h^3^-FNCs exhibit excellent oxidase-like activity compared to the reference samples, with its mass activity and metal mass-specific activity being respectively 66 and 315 times higher than those of commercial 30% Pt/C catalysts (Fig. 4c, d). In particular, h^3^-FNCs have the highest catalytic rate constant (K_cat_) compared to the other catalysts (Fig. S8e and Table S4). Notably, the metal mass-specific activity of h^3^-FNCs is 2.3 times higher than those of m-FNCs and l-FNCs. A number of studies suggest that neighboring sites can modify the electronic structure to influence catalytic activity [34, 35]. Hence, the much-enhanced metal mass-specific activity of h^3^-FNCs may be attributed to the synergy between neighboring sites by a density effect. Specifically, when the distances between Fe sites decrease to approximately 4 Å (Fig. 2f) by elevated iron loading, adjacent Fe–N_4_ moieties will robustly and inevitably interact with each other, refining their electronic structure and thereby enhancing the intrinsic oxidase-like activity. Finally, myriad sites with density effect will substantially boost the apparent oxidase-like activity of h^3^-FNCs. To the best of our knowledge, h^3^-FNCs exhibit the highest mass activity among the oxidase-like catalysts reported in the literatures (Table S5). According to Fig. S8f, the catalytic activity of h^3^-FNCs does not exhibit a linear dependence on the pH of the solution, and the observed slight reduction in TMB consumption may be ascribed to the different solubilities of TMB in different pH solutions [7]. This pH-independent characteristic endows h^3^-FNCs with the potential for applications in various pH conditions. And h^3^-FNCs exhibit catalase-like activity (CAT) and better oxidase-like activity under the presence of H_2_O_2_, suggesting the potential advantages of h^3^-FNCs for applications in physiological environments containing H_2_O_2_ (Fig. S9).

In addition, the catalytic performance of h^3^-FNCs shows almost no decay after half a year of storage in deionized water at room temperature (Fig. 4e), indicating their extremely high stability. Such a high stability is proposed to stem from the strong binding between the coordinated Fe atoms and the support surface, and matrix surface lattice remodeling by Fe atoms [12, 36]. The result of precipitation of Fe in different solutions measured by inductively coupled plasma mass spectrometry (ICP-MS) also indicates Fe single atoms in h^3^-FNCs exhibit high stability and will not leach into the solution (Table S6). Furthermore, the aqueous stability of h^3^-FNCs was evaluated by DLS measurements (Fig. S10). The results indicate that h^3^-FNCs remained stable over 72 h and exhibited good dispersion in physiological environment such as DI water, PBS and culture medium. In contrast to common antimicrobial drugs, the excellent storage stability of h^3^-FNCs offers significant advantages for transportation, storage and usage, benefiting future clinical translation.

To further elucidate the catalytic activity of h^3^-FNCs, we employed electron paramagnetic resonance (EPR) to detect possible active intermediates, using DMPO as a spin-trapping agent. As shown in Fig. 4f, in the presence of h^3^-FNCs, the EPR signal clearly shows six characteristic peaks with relative peak intensity ratios of 1:1:1:1:1:1, which can be attributed to hydroperoxyl radicals (·OOH, formed by the transformation of O_2_·^−^), confirming the catalytic production of O_2_·^−^ [37]. The variation of peak intensities demonstrates that h^3^-FNCs are more efficient in catalyzing ROS production than the commercial Pt/C and FNCs with lower metal loadings, highlighting the positive correlation between catalytic activity and the density of single-atom sites. Then, we used the fluorescent probe dihydroethidium (DHE) to further corroborate the generation of O_2_·^−^ during the catalytic reaction. The results (Fig. 4g) show the introduction of h^3^-FNCs enhances the fluorescence intensity, signifying the oxidation of DHE. Notably, the fluorescence intensity reaches its maximum after the addition of GSH, demonstrating that GSH is able to accelerate the DHE oxidation process mediated by h^3^-FNCs. In addition, the catalytic depletion of GSH by h^3^-FNCs was analyzed using a DTNB-GSH assay kit, and the results are depicted in Fig. S11. The reaction mechanism between DTNB and GSH is shown in the following Eqs. (1–3):

1 $${\text{O}}_{2} + e^{ - } \to ^{{h^{3} - {\text{FNC}}}} {\text{O}}_{2}^{ \cdot - }$$

2 $$2{\text{GSH}} + {\text{O}}_{2}^{ \cdot - } + 2{\text{H}}^{ + } \to ^{{{\text{h}}^{3} { - }{\text{FNC}}}} {\text{GSSG}} + 2{\text{H}}_{{2}} {\text{O}}$$

3 $${\text{DTNB}} + 2{\text{GSH}} \to {\text{GSSG}} + 2{\text{TNB}}$$

In Fig. 4h, the presence of h^3^-FNCs (3 ppm) in a GSH-containing solution significantly influences the GSH oxidation. As shown in Fig. 4i, the peak intensity of TNB at 420 nm decreases with the increase in reaction time. The characteristic peak of TNB completely disappears after the reaction for 30 min, proving that the GSH in the solution has been completely consumed. Given that GSH is a primary redox substance for maintaining metabolism and homeostasis in bacteria, this catalytic property of h^3^-FNCs lays a solid foundation for the subsequent antibacterial treatment.

### Density Functional Theory Calculations

**Fig. 5:**
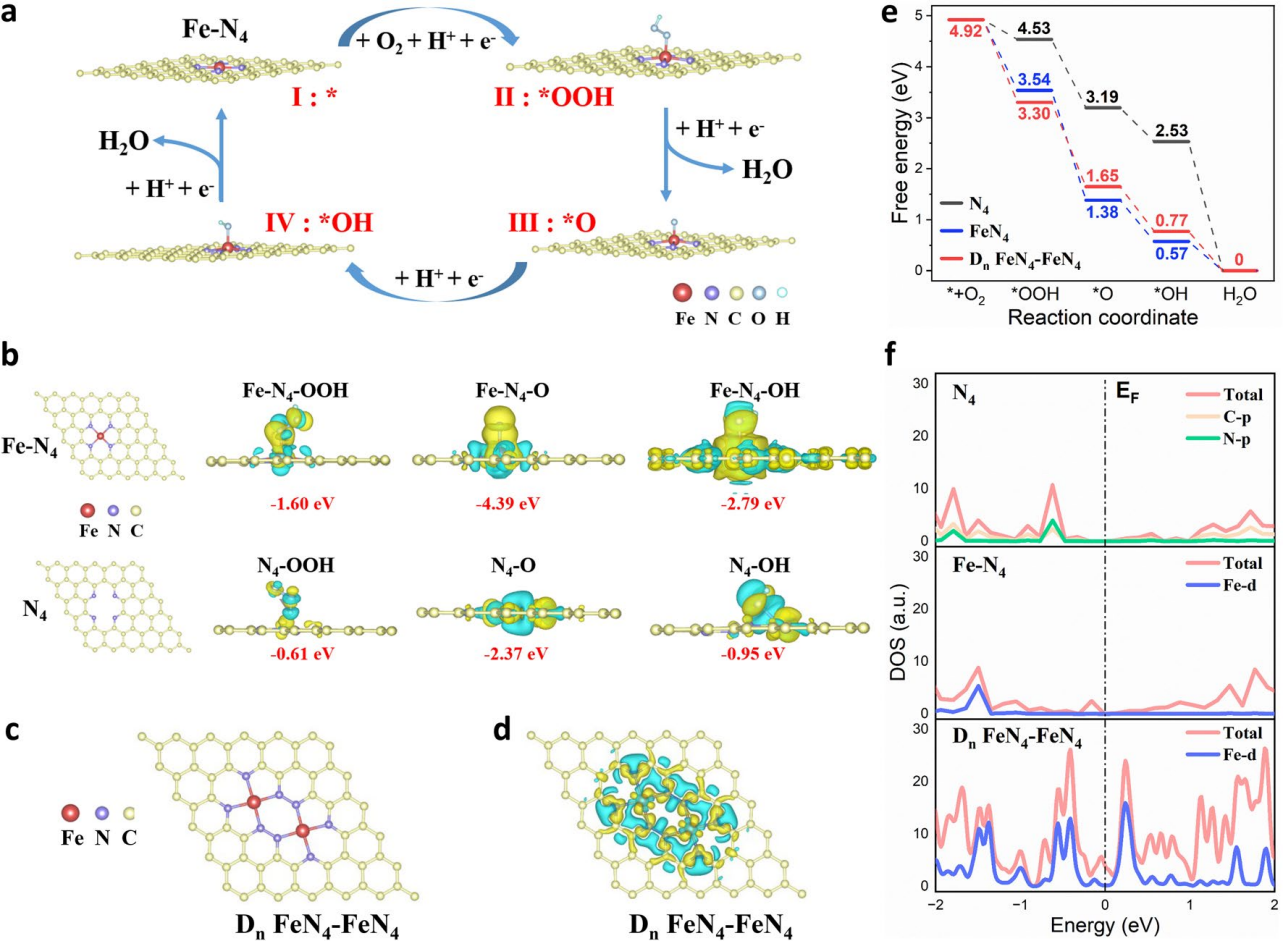
DFT calculations of the oxidase-like activity. **a** Proposed reaction pathways of O_2_ activation on h^3^-FNCs. The red, purple, yellow, dark blue and baby blue balls represent the Fe, N, C, O and H atoms, respectively. **b** Optimal configurations of Fe–N_4_ or N_4_ by VASP, and the charge density difference plots for reaction intermediates (*OOH, *O, *OH) interacting with Fe–N_4_ or N_4_. The negative values are the adsorption energy (E_ad_). The yellow and blue colors refer to the accumulation and depletion of electrons, respectively. The value of charge transfer is also denoted in the supplementary information (Table S7). **c** Optimal configuration of D_n_ FeN_4_–FeN_4_. **d** Top views of the charge density difference plots of D_n_ FeN_4_–FeN_4_. **e** Free energy profiles of O_2_ activation at N_4_, Fe–N_4_, and D_n_ FeN_4_–FeN_4_ site models (U = 0 V, T = 298 K and P = 1 bar). **f** Density of states of N_4_, Fe–N_4_ and D_n_ FeN_4_–FeN_4_ with the E_F_ level aligned at 0 eV

The mechanism behind the high oxidase-like activity of h^3^-FNCs was deciphered by DFT calculations. We constructed a model of the Fe–N_4_ active center and simulated its catalytic reaction process. As depicted in Fig. 5a, the four-electron reaction pathway commences with the adsorption and protonation of O_2_ molecules at the Fe–N_4_ sites, forming *OOH species. Subsequently, *OOH combines with H^+^ and dissociates into activated *O species and H_2_O. The *O is then protonated to yield *OH, which further reacts with H^+^ to form adsorbed H_2_O molecules at the active sites. Finally, the H_2_O desorbs and catalyst re-exposes the active sites for the next cycle of reaction. Bader charge was then obtained at each stage of the four-electron reaction to reveal the intrinsic origins for the high catalytic activity of Fe–N_4_. Figure 5b and Table S7 present the detailed adsorption energy (*E*_ad_) changes and electron transfer during the reaction, revealing that more electron transfer corresponds to the stronger adsorption of the intermediates on the active sites and the intensified interactions between them. The electron cloud accumulation and dissipation patterns indicate that the intermediates are electron acceptors while the active sites are electron donors. The adsorption energy levels of Fe–N_4_ for all intermediates are higher than that of N_4_, suggesting a much more favorable adsorption of intermediate species by Fe–N_4_ than N_4_.

Numerous proximate Fe sites are present in h^3^-FNCs, which exhibit enhanced intrinsic site activity. To reveal the synergistic catalytic effect between neighboring Fe atoms and their advantages over isolated single-atom sites, we established a model featuring neighboring FeN_4_–FeN_4_ sites (D_n_ FeN_4_–FeN_4_) embedded in the lattice of graphene surface (Fig. 5c), and the Fe–Fe distance is set at 4.0 Å, based on experimental measurements (Fig. 2f). The desorption energy of *OH from single Fe atom sites generally serves as an indicator of ORR activity [38]. As shown in Fig. 5d, the number of electrons transferred from a single Fe atom in D_n_ FeN_4_–FeN_4_ is 1.068 e, whereas, the number is 1.395 e in FeN_4_. The fewer electrons transfer of Fe results in the weaker *OH adsorption, which leads to easier *OH detachment and higher catalytic activity. To verify this interpretation, we further calculated the free energies of oxidase-like reactions catalyzed by N_4_, FeN_4_, and D_n_ FeN_4_–FeN_4_ respectively. The Gibbs free energy diagram (Fig. 5e) identifies the last step as the rate-determining step (RDS) for Fe–N_4_ and D_n_ FeN_4_–FeN_4_, while the first step is the RDS for N_4_. The decreasing free energy of all reaction steps indicates that the catalytic reaction is thermodynamically feasible at room temperature [39], which is consistent with the high catalytic activity from the experimental outcomes. In contrast to 0.84 V for N_4_, the overpotential of FeN_4_ is estimated to be 0.66 V (Table S8). Furthermore, the overpotential of D_n_ FeN_4_–FeN_4_ is 0.46 V, which is much lower than that of FeN_4_. Such a substantial decrease in the overpotential, due to the reduced *OH binding energy, suggests a distinct enhancement in the catalytic activity of D_n_ FeN_4_–FeN_4_, which is consistent with previous calculations of the Bader charge. The initial electron transfer to the adsorbed O_2_ to form O_2_·^−^ is a critical step in ORR. To further clarify the actual O_2_·^−^ generation catalyzed by different configurations, we used the oxygen adsorption model for simulation. The adsorption energy level and the electron transfer (Fig. S12) indicate that D_n_ FeN_4_–FeN_4_ exhibits the best O_2_ adsorption accessibility and more electron transfer to O_2_, which reflects a greater capacity of D_n_ FeN_4_–FeN_4_ for oxygen activation and O_2_·^−^ production compared to N_4_ or Fe–N_4_.

In addition, the density of states (DOS) was calculated to reveal the electronic properties of all the models. As shown in Fig. 5f, the Fe–N_4_ structure possesses a higher DOS at the Fermi energy (*E*_*F*_) level and a relatively strong Fe peak around − 1.5 eV, indicating that Fe single atom embedding enhances the electrical conductivity of the system and accelerates the charge transfer in the reaction, in agreement with previous findings [40]. The DOS at *E*_F_ level is apparently higher in *D*_n_ FeN_4_–FeN_4_ than in FeN_4_, suggesting that FeN_4_–FeN_4_ sites are more sensitive to reaction intermediates’ adsorption or desorption. This outcome may be attributed to the reduced distance between two adjacent Fe atoms, leading to the downshifted energy of the electron orbitals, reduced on-site spin moment and weakened binding strength of the *OH adsorption [41, 42]. These results further verify the distinguished advantage of electronic structure modulation by the proximate atoms. In conclusion, DFT calculations and the experimental results validate that the density effect originating from the interaction between the adjacent single Fe atoms is responsible for the substantially enhanced catalytic activity of h^3^-FNCs.

### In Vitro Antimicrobial Effect Evaluation

#### Antibacterial Activity of h^3^-FNCs in Vitro

**Fig. 6:**
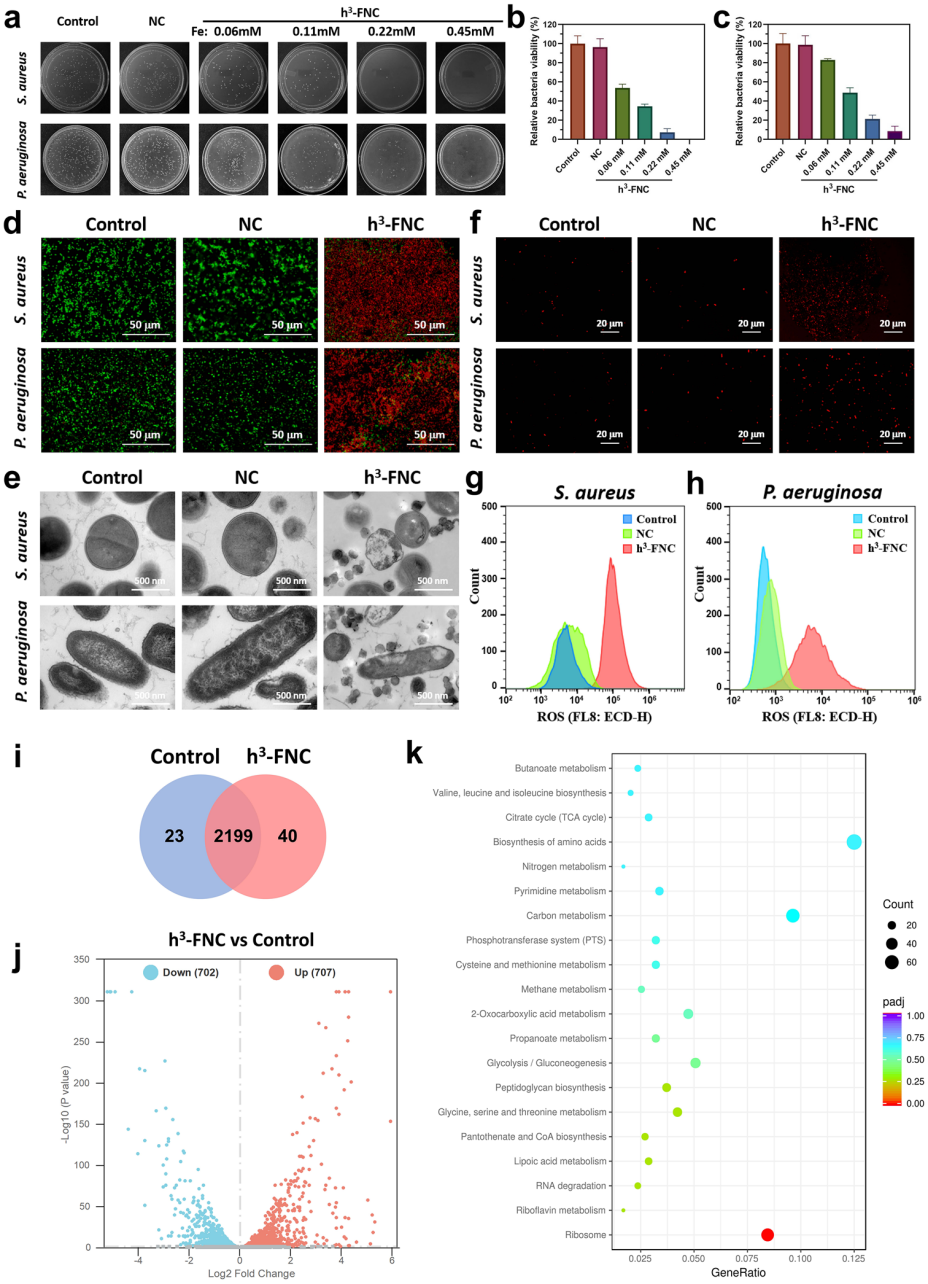
In vitro antibacterial activity of h^3^-FNCs. **a** Photo-images of *S. aureus* and *P. aeruginosa* colonies post-treatment with saline, NCs and different concentrations of h^3^-FNCs. **b, c** Relative viabilities of *S. aureus* and *P. aeruginosa* based on the colony images in **a**. **d** Confocal images of live/dead stained *S. aureus* and *P. aeruginosa* under different treatments. **e** TEM images of *S. aureus* and *P. aeruginosa* after different treatments. **f** Fluorescence images depicting intracellular ROS levels in *S. aureus* and *P. aeruginosa* by DHE staining. **g, h** Flow cytometric analysis of DHE stained bacteria for the evaluation of ROS levels in *S. aureus* and *P. aeruginosa*. **i** Venn diagram of overlapping significantly changed genes (*P* < 0.05). **j** Volcano plot for the distribution of DEGs in h^3^-FNC compared with control. **k** KEGG enrichment of top 20 relevant pathways in response to h^3^-FNC

h^3^-FNCs exhibit much-enhanced oxidase-like activity and ROS production efficiency, thus having the potential to trigger oxidative stress and damage bacteria [43]. To thoroughly assess the antimicrobial activity of h^3^-FNCs, we selected two primary healthcare-associated infection pathogens: Gram-positive *Staphylococcus aureus* (*S. aureus*) and Gram-negative *Pseudomonas aeruginosa* (*P. aeruginosa*) for in vitro assays. Initial assessments of bacterial survival were conducted using the plate count method and live/dead staining techniques. As shown in Fig. 6a–c, only a slight reduction in the bacterial count of the control and NC groups can be seen, in contrast, the colony numbers of *S. aureus* and *P. aeruginosa* show remarkable decreases at increased concentrations of h^3^-FNCs. The live/dead staining images (Fig. 6d) predominantly show red fluorescence in the presence of h^3^-FNCs, indicating that bacteria are dead or their membranes are largely damaged. This finding is consistent with the bacterial SEM images (Fig. S13 and TEM images in Fig. 6e), where bacterial membranes are severely damaged and their morphologies are largely abnormal. These antibacterial experiments demonstrate that h^3^-FNCs are highly active in killing bacteria, which can be mainly attributed to the intrinsic catalytic activity. Meanwhile, a CCK-8 assay was used to verify the cytocompatibility of h^3^-FNCs in vitro. The results (Fig. S14) demonstrated the safety of h^3^-FNCs (400 ppm) exposure to human skin fibroblasts (HSF). To further identify the role of their superior oxidase-like activity in the antibacterial process, we measured the intracellular ROS levels in bacteria after co-incubation with the material. Dihydroethidium (DHE), a cell-permeable fluorogenic probe, was employed to quantify the intracellular ROS. As shown in Fig. 6f, a strong fluorescent signal is detected in the h^3^-FNCs group, demonstrating the burst of ROS within the bacteria. Flow cytometric analysis (Fig. 6g, h) further proves that h^3^-FNCs can significantly elevate the ROS levels in bacteria. Furthermore, a microplate dilution assay was performed to ascertain the minimum inhibitory concentration (MIC) of h^3^-FNCs, and the MIC values against *S. aureus* and *P. aeruginosa* were 40 and 80 µg mL^−1^, respectively (Fig. S15). It is noteworthy that *P. aeruginosa* is more resistant to oxidative stress damage than *S. aureus,* possibly due to the additional lipid membrane present in the cell walls of Gram-negative bacteria [44]. Consequently, the bactericidal effect of h^3^-FNCs is more pronounced against *S. aureus* than against *P. aeruginosa*, which also echoes the colony counting results of the initial antibacterial assays. In conclusion, h^3^-FNCs are capable of efficiently catalyzing ROS production in vitro to directly destroy bacteria, thus circumventing potential bacterial resistance and exhibiting favored broad-spectrum antibacterial activity.

#### Antibacterial Mechanism of h^3^-FNCs

To elucidate the comprehensive antibacterial mechanism of h^3^-FNCs against *S. aureus*, RNA sequencing analysis was conducted. The resulting Venn diagram and volcano plot indicated that among 2,262 expressed genes in both groups, 702 genes were down-regulated and 707 genes were up-regulated in the h^3^-FNC group compared with the control (Figs. 6i, j and S16). Gene Ontology (GO) enrichment analysis revealed differentially expressed genes (DEGs) related to carbohydrate metabolism, membrane transport systems, lipid metabolism, translocation and energy metabolism (Fig. S17). Furthermore, Kyoto Encyclopedia of Genes and Genomes (KEGG) pathway enrichment analysis shows that treatment with h^3^-FNCs significantly impacted several metabolic pathways on *S. aureus*, including the biosynthesis of various secondary metabolites, citric acid cycle (TCA cycle), biosynthesis of amino acids, GSH metabolism, pyrimidine metabolism and RNA degradation (Fig. 6k). These pathways are integral to critical regulatory systems governing the bacterial life cycle. Consequently, it can be inferred that the antibacterial activity of h^3^-FNCs is closely associated with the oxidase-like activity to catalyze ROS production for destroying bacterial membranes and GSH consumption for disrupting metabolic processes, which exert direct damage to the bacteria, including their intracellular components and various essential cellular processes.

### In Vivo Antimicrobial Therapy Assessment

**Fig. 7:**
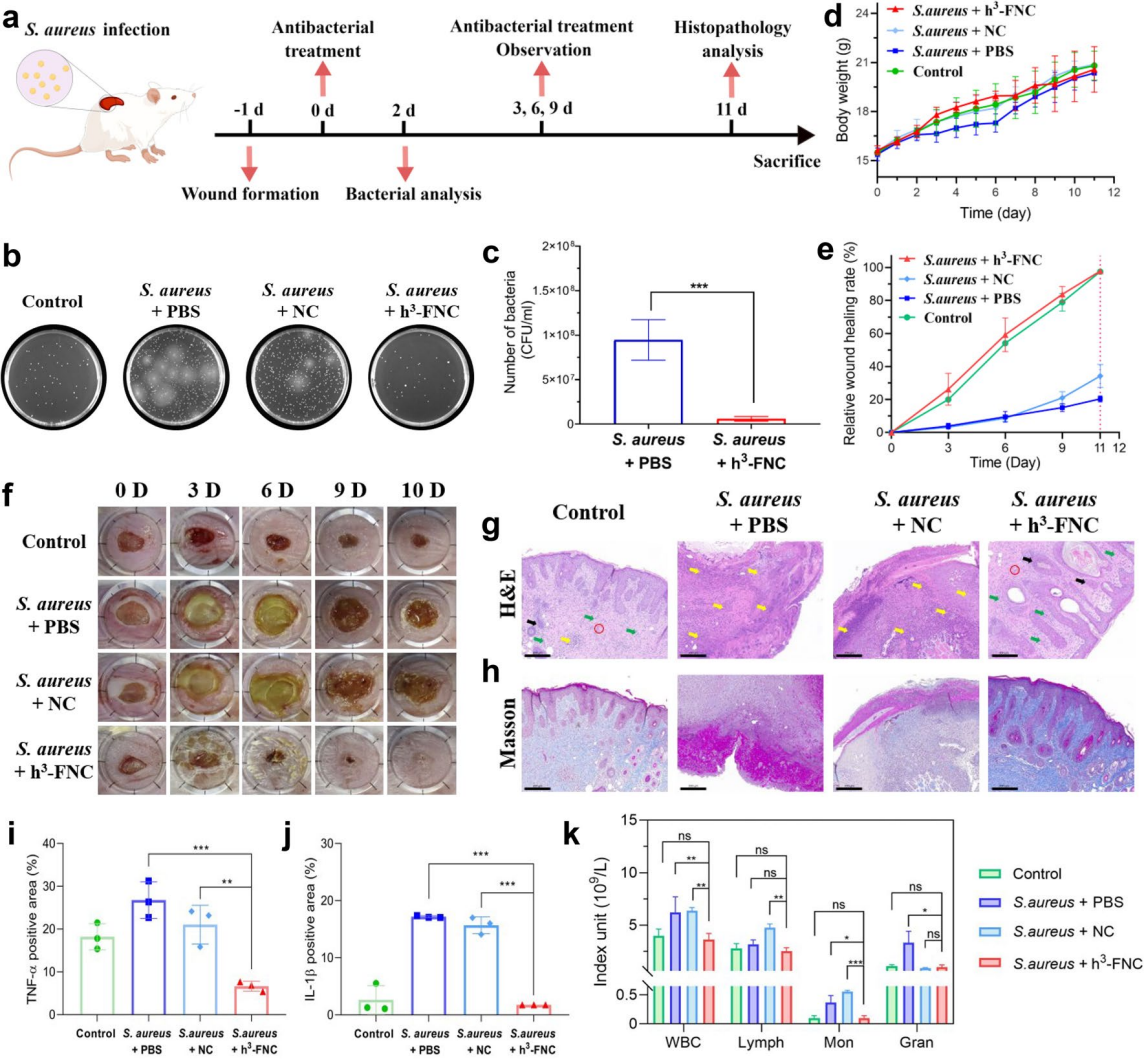
In vivo antibacterial performance of h^3^-FNCs.** a** Schematics of treatment strategy in the *S. aureus*-infected mouse wound model (drawn by Figdraw). **b** Images of bacterial colonies separated from the wound tissue plated on LB agar plates in 2 days of treatment. **c** Quantities of surviving bacteria in the wound tissues of two groups (*S. aureus* + PBS and *S. aureus* + h^3^-FNCs) in 2 days of treatment. **d** Body weight changes of different groups during the wound healing process. **e** Relative wound healing rate of mice after various treatments for varied treatment intervals. **f** Representative photographs of uninfected wounds and *S. aureus*-infected wounds treated with PBS, NC and h^3^-FNCs on days 0, 3, 6, 9, and 10. The diameter of the circular ruler is 12 mm. **g** H&E and **h** Masson staining of the bacteria-infected tissues after different treatments. Yellow arrows reflected inflammatory cells, including macrophages, lymphocytes, and neutrophils. Green and black arrows represent fibroblasts and hair follicles, respectively. Red circles represent neovascularization. Scale bar: 200 μm. **i, j** Quantitative analysis of TNF-α and IL-1β expression of different groups. **k** Inflammatory cell numbers in the blood of mice in different groups. Statistical analysis was performed using the two-tailed t-test. Data represent means ± SD, *n* = 6. ***p < 0.001, **p < 0.01, *p < 0.05

Encouraged by the excellent antibacterial activity of h^3^-FNCs in vitro, the antimicrobial efficacy in vivo was evaluated by an *S. aureus*-infected mouse wound model (Fig. 7a). Firstly, the healthy female BALB/c mice were randomly divided into 4 groups: the control group with wound incisions without *S. aureus* infection (Control), the untreated group with infected wounds without any treatment (*S. aureus* + PBS), and the group treated with NCs (*S. aureus* + NC) or h^3^-FNCs (*S. aureus* + h^3^-FNC) as the therapeutic agent. To establish an infection model, circular wounds of approximately 8 mm in diameter were created on the dorsal area of mice and then injected with 10 μL of 1 × 10^8^ CFU/mL *S. aureus*. Adhering to the clinical medical concept of “safe and effective, simple and reliable” [45], we directly applied h^3^-FNCs to the *S. aureus*-infected wounds externally.

#### Antibacterial Effect of h^3^-FNCs in Vivo

The intracellular ROS levels within the wound 2 h after first administration were monitored by fluorescence staining to verify the oxidase-like ability of h^3^-FNCs (Figs. S18 and S19). A dramatically enhanced red fluorescence at the debris was observed in h^3^-FNC group compared to the other groups, indicating the strong ability of h^3^-FNCs to catalyze ROS production for bacterial killing. After treatment for 2 days, wound tissue was harvested and transferred to an agar plate for incubation to quantify the survival of bacterial colonies. As shown in Figs. 7b and S20, the treatment with h^3^-FNCs could evidently reduce the number of bacteria remaining in the wounds compared to the other *S. aureus*-infected groups. Based on the bacterial counting results (Fig. 7c), the *S. Aureus* + h^3^-FNCs group exhibits significantly fewer colonies than the *S. Aureus* + PBS group, indicating the excellent bactericidal effect of h^3^-FNCs. After 4 days of initial administration, the tissues of mice were collected for ICP analysis and the result shows that h^3^-FNCs leave no residues in visceral or skin wound tissues (Fig. S21). And during the treatment period, there were no significant fluctuations in body weight of the treatment groups, indicating the good biocompatibility of h^3^-FNCs (Fig. 7d). Notably, the mice exhibit obvious ulcerated and pus-filled wounds after S. aureus injection, but those treated with h^3^-FNCs show a marked and progressive reduction in wound size (Fig. 7e, f). Moreover, the infectious wounds treated with h^3^-FNCs display no edema and form scabs in three days, followed by complete healing in about 10 days of therapy, with an average infected wound healing rate of 97.8%, revealing the excellent antibacterial efficacy of h^3^-FNCs.

#### Safety Analysis of h^3^-FNCs in Mice

Then, hematoxylin and eosin (H&E) and Masson staining were used to monitor the wound healing process. From the H&E staining results (Fig. 7g), h^3^-FNCs group hardly displays inflammatory cells in wound areas while numerous inflammatory cells can be found in the other *S. aureus*-infected groups. Histological examination reveals that keratin-forming cells have migrated from normal tissues to the wound site, collagen (stained blue) deposition has increased, and the skin epidermis of the wound has thickened and become completely cured after the h^3^-FNCs treatment (Fig. 7h). These findings align with the immunohistochemical (IHC) staining results for tumor necrosis factor-alpha (TNF-*α*) and interleukin-1 beta (IL-1*β*), showing significantly lower expression levels of these inflammatory factors in the control and h^3^-FNC group compared to PBS and NC groups (Figs. 7i, j and S22). In addition, biochemical index assessments and pathological analyses of major organs (heart, liver, spleen, lungs and kidneys) were performed to investigate the long-term toxicity of h^3^-FNCs in vivo. In comparison to other groups, blood biochemical indicators such as monocytes (Mon), lymphocytes (Lymph) and white blood cells (WBC) in mice treated with h^3^-FNCs return to normal levels (Fig. S23). And the group treated with h^3^-FNCs demonstrates a markedly lower level of blood inflammatory cells compared to the other bacterial-infected groups (Fig. 7k), indicating the inflammation reduction by the potent antibacterial performance of h^3^-FNCs. Furthermore, there is no significant damage, inflammation or abnormalities observed in the heart, liver, spleen, lung or kidney tissues of the mice treated with h^3^-FNCs, validating the high biocompatibility of h^3^-FNCs with minimal toxic side effects in mice (Fig. S24). Therefore, the results above indicate that h^3^-FNCs are promising safe and efficient candidates for the treatment of bacterial infections in vivo.

## Conclusions

In summary, an ion exchange approach has been successfully developed to synthesize especially high Fe-loading single-atom catalysts, which feature high metal loading, ultrahigh catalytic activity and high stability for efficient and convenient antibacterial therapy. A density effect underlying the ultrahigh oxidase-like activity of h^3^-FNCs has been identified and elucidated through diversified material characterizations, rigorous catalytic performance tests and DFT theoretical calculations, which reveals the important role of metal density modulation and the interaction between two adjacent Fe atoms in the close enough vicinity in endowing h^3^-FNCs with especially high catalytic performance. The present high Fe-loading SACs featuring density effect demonstrate the greatly enhanced and long-lasting oxidase-like activity for medical purposes compared with literature-reported SACs, confirming the link between density regulation and catalytic activity changes of active sites. Both in vitro and in vivo assays demonstrate the impressive broad-spectrum bactericidal performance of h^3^-FNCs, with an average complete wound healing rate of 97.8% and favorable biosafety. In addition to the antibacterial therapy, the present h^3^-FNCs are expected to offer great potential in the treatment of a number of diseases, such as tumors and viruses [46, 47]. The synthetic method may provide an alternative approach to the design of catalysts for catalyzing reactions necessitating the synergistic interaction of multiple sites, such as C–C coupling [48]. These findings present a highly attractive strategy to increase the metal loading of SACs and the intrinsic activity of single active sites.

## Supplementary Information

Below is the link to the electronic supplementary material.

Supplementary file1 (DOCX 14398 kb)
