# Supplementary material for: High Fe-Loading Single-Atom Catalyst Boosts ROS Production by Density Effect for Efficient Antibacterial Therapy
Source: Nanomicro Lett. 2024 Oct 4;17:32. doi: 10.1007/s40820-024-01522-1 (PMC11450126; doi:10.1007/s40820-024-01522-1)
Supplement: Supplementary file 1 — Supplementary file1 (DOCX 14398 kb) [file 40820_2024_1522_MOESM1_ESM.docx]

Supporting Information for

**High Fe-Loading Single-Atom Catalyst Boosts ROS Production by Density Effect for Efficient Antibacterial Therapy**

Si Chen^1, 2^, Fang Huang^1^, Lijie Mao^1^, Zhimin Zhang^2^, Han Lin^2^, Qixin Yan^1^, Xiangyu Lu^1, 2,^ *, Jianlin Shi^1, 2,^ *

^1^Shanghai Tenth People's Hospital, Shanghai Frontiers Science Center of Nanocatalytic Medicine, School of Medicine, Tongji University, Shanghai, 200092, P. R. China

^2^Shanghai Institute of Ceramics, Chinese Academy of Sciences, Shanghai, 200050, P. R. China

*Corresponding authors. E-mail: [jlshi@mail.sic.ac.cn](mailto:jlshi@mail.sic.ac.cn) (Jianlin Shi), [xiangyulu@126.edu.cn](mailto:xiangyulu@126.edu.cn) (Xiangyu Lu)

**S1 Supplementary Methods**

**S1.1 XAFS measurements and simulations**

The XAFS data of the absorption spectra of Fe K-edge were collected in transmission mode at room temperature using a Si (111) double crystal monochromator at BL14W1 station of Shanghai Synchrotron Radiation Facility (SSRF). The recorded XAFS data were background-subtracted and normalized in Athena (version 0.9.26).

For Wavelet Transform analysis, the χ(k) exported from Athena was imported into the Hama Fortran code. The parameters were listed as follows: R range, 1 – 4 Å, k range, 0 – 10 Å^-1^; k weight, 2; and Morlet function with κ=10, σ=1 was used as the mother wavelet to provide the overall distribution [S1].

**S1.2** **Mössbauer measurements**

The Mössbauer measurements were performed at room temperature using a conventional spectrometer (Germany, Wissel MS-500) in transmission geometry with constant acceleration mode. A _57_Co (Rh) source with an activity of 25 mCi was used. The velocity calibration was done with a room temperature α-Fe absorber [S2]. The spectra were fitted by the software Recoil using Lorentzian Multiplet Analysis.

**S1.3 Catalytic analysis**

Oxidase activities were determined by colorimetric assays. TMB could be oxidized by ROS to ox-TMB with a characteristic absorption peak at 652 nm. We added h^3^-FNCs (0~4 ppm) into 1.0 mL DI water containing different concentrations of TMB (0~2 mM) and recorded the absorption peak at 652 nm of the ox-TMB for activity testing. The catalytic efficiency was studied by measuring the absorption changes of ox-TMB at 652 nm (ε = 39,000 M^-1^·cm^-1^). The activities of m-FNC, l-FNC and commercial Pt/C were also tested for comparison. The Michaelis-Menten constant was calculated using Lineweaver-Burk plots of the double reciprocal of the Michaelis-Menten equation:

$$v=\frac{v_{max}\times[S]}{K_{m}+[S]}$$

where v is the initial velocity, v_max_ is the maximal reaction velocity, [S] is the concentration of substrate, and K_m_ is the Michaelis constant.

The mass activity is determined by dividing the catalytic velocity by the mass concentration of the catalyst, and the metal mass-specific activity is calculated by dividing the catalytic velocity by the metal mass concentration of the catalyst. To detect O_2_•^-^, DMPO (25 mM) and h^3^-FNCs (50 ppm) were added into 0.1 mL methanol solution, and the mixture was vortexed vigorously for 30 seconds and then incubated for 5 minutes to record the EPR spectra. DHE is a common fluorescence detection probe for ROS, which can be oxidized to ethidium (ox-E) in the presence of O_2_•^-^. The oxidation of DHE was characterized by fluorescence spectroscopy recording the fluorescence peak of ox-E (excitation wavelength 520 nm, emission wavelength 625 nm) to study the oxidase-like activity of the catalyst. In this test, h^3^-FNCs (20 ppm) and DHE (100 μM) were added into DI water (3 mL) with or without GSH (300 μM), and the fluorescence spectra were then recorded after 20 minutes of reaction. h^3^-FNCs (5 ppm) and GSH (250 μM) were mixed in 1 mL of phosphate buffered salt solution (PBS, pH 7.4) and incubated for 10, 20 and 30 min, respectively, according to the method of DTNB-GSH assay kit from Beyotime. Then DTNB was added and after 3 minutes of reaction, the absorbance of the mixed solution at 412 nm was measured by a microplate spectrophotometer. To further investigate the depletion of GSH, h^3^-FNCs (10 ppm) and GSH (300 μM) were mixed in 1 mL of PBS and incubated for 5, 10 and 30 minutes respectively. We recorded the absorbance of the mixed solutions with a UV spectrophotometer based on the same DTNB assay described above.

**S1.4 DFT calculation details**

DFT calculations in this study were performed by employing the Vienna ab initio simulation package (VASP) [S3] to reveal the intrinsic catalytic mechanism of h^3^-FNC. To optimize and determine the free energy of all structures, the projected enhancement wave pseudopotential [S4] and the generalized gradient approximation (GGA) of Perdew, Burke and Enzzerhof (PBE) exchange-correlation functional [S5] are applied in the program. While the cutoff energy of the plane waves basis set was 500 eV, a Monkhorst-Pack mesh of 2×2×1 was used in K-sampling in the adsorption energy calculation. For the process of geometry optimization, the electronic self-consistent iteration was set to 10^−5^ eV, and the locations of all atoms were totally relaxed until the force on each atom reduced below 0.05 eV Å^-1^. The distance of the vacuum layer was set as 15 Å along the z-direction to prevent periodic interactions.

To comprehend the enhanced ORR catalytic behavior by electronic interaction of adjacent Fe–N_4_ moieties, the projected augmented wave (PAW) potentials [S4] were chosen to describe the ionic cores with a kinetic energy cutoff of 520 eV. The Gaussian smearing method and a width of 0.2 eV were employed to allow partial occupancies of the Kohn−Sham orbitals. The max force of 0.02 eV Å^−1^ was set as the convergence standard for geometry optimization. The vacuum distance was set to 18 Å for spurious interaction exclusion. The weak interaction was described by DFT+D3 method with empirical correction in Grimme’s scheme [S6].

Conventionally, the reaction pathway by which h^3^-FNCs catalyze ROS production was considered as the following elementary steps:

The asterisk (*) is the active site of the catalyst. The free energy change (Δ*G*) calculations of each elementary reaction were computed by the following equation (5):

Δ*G* = Δ*E* + ∆*ZPE* - *T*∆*S* (5)

Where Δ*E*, ∆*ZPE*, T, and ∆*S* are the reaction energy difference, zero-point energy change, temperature, and entropy change, respectively.

**S1.5 Bacteria culture**

*Staphylococcus aureus* (*S. aureus*) and *Pseudomonas aeruginosa* (*P. aeruginosa*) were chosen as representatives of Gram-positive and negative bacteria, respectively. These bacteria are the main flora causing human diseases. The bacteria were cultured in LB broth at 37 °C (240 rpm shaking) and passaged every day.

**S1.6 In vitro antibacterial assays**

The bacteria (*S. aureus* and *P. aeruginosa*) were incubated overnight in LB medium at 37 ℃. After passage in LB, bacteria of logarithmic growth phase (OD_600_=0.5, 10^8^ Colony Forming Units (CFU) / mL) were incubated with NCs, different concentrations of h^3^-FNCs (Fe concentration: 0.06, 0.11, 0.22, 0.45 mM, respectively) in saline for 4 hours at 37 ℃. Then, the bacterial suspensions were diluted and spread evenly on LB agar plates for 19 hours at 37 ℃. After incubation, the corresponding bacterial colonies were counted to quantify bacteriostasis. All experiments were repeated 3 times, and the bacterial viability was calculated by the ratio of the number of colonies formed in the experimental group to that formed in the blank control group.

***S1.6.1 Live/dead staining test***

To obtain more details on bacterial viability, the bacteria after the different treatments above were collected and stained with SYTO 9 and propidium iodide (PI) in the dark at 37 ℃ for 30 minutes, followed by washing 3 times using saline solution. The live (green fluorescence) and dead (red fluorescence) images of bacteria were observed by fluorescence microscopy.

***S1.6.2 Cellular ROS assay***

To investigate the ROS generation in bacteria, the bacteria were collected and stained with DHE (10 µM) in the dark at 37 ℃ for 20 minutes, followed by incubation with NCs and h^3^-FNCs (200 ppm) in saline for 30 minutes. Then, the mixture solutions were washed 3 times and resuspended with saline for observation by fluorescence microscopy and flow cytometry.

***S1.6.3 Morphological observation***

For **SEM** images, bacterial samples treated with different materials were collected, centrifuged at 8000 rpm for 3 minutes and resuspended in 2.5% glutaraldehyde solution in the dark for 12 hours at 4°C. The fixed samples were rinsed 3 times in PBS buffer (0.1 M, pH=7.4) and then fixed in 1% osmium tetroxide in the dark for 1 hour at room temperature. The samples were further sequentially dehydrated in ethanol solution with increasing concentration (30%, 50%, 70%, 80%, 90%, 100%) and 100% isoamyl acetate for 15 minutes each. After drying, the cell samples were observed by a scanning electron microscope.

For **TEM** characterization, bacterial samples after various treatments were fixed in 2.5% glutaraldehyde solution in the dark for 12 h. After washing with PBS, the suspension samples were pre-embedded in 1% agarose solution and then in 1% osmium tetroxide in the dark for 2 hours at room temperature. The samples were further sequentially dehydrated in ethanol solution with increasing concentration (30%, 50%, 70%, 80%, 90%, 95%, 100%) and 100% acetone for 20 minutes each. The samples were permeabilized with mixed acetone and 812 embedding agent at 37°C, polymerized at 60 °C for 48 h and then stained in sections (2.6% lead citrate solution) and finally observed by transmission electron microscopy.

**S1.7 In vivo catalytic antibacterial therapy**

All the animal procedures were performed in compliance with the guidelines of the Regional Ethics Committee for Animal Experiments approved by the Administrative Committee of Laboratory Animals of Shanghai Tenth People’s Hospital, Tongji University School of Medicine (SHDSYY-2023-21162). Healthy female BALB/c mice from 6 to 8 weeks of age were provided by SPF Biotechnology Co. Ltd (Beijing, China). Prior to treatment, all animals were housed under standard conditions for 7 days (temperature 20~25 °C, 12-hour light/dark cycle, humidity of 60~70%). Animals were randomly divided into the following 4 groups (6 mice in each group): (1) the control group with uninfected wounds (Control), (2) the untreated group with infected wounds without any treatment (*S. aureus* + PBS), (3) the material control group treated with NCs as the therapeutic agent (*S. aureus* + NC), and (4) the material group treated with h^3^-FNCs as the therapeutic agent (*S. aureus* + h^3^-FNC). To establish a trauma model, all mice were debrided and a circular incision (8 mm in diameter) was excised on the back of each mouse. For the infected wound model mice (Group 2~4), 10 μL *S. aureus* suspension (1×10^8^ CFU/mL) was injected into the dorsal flank skin within the wound region. After 24 hours of infection, 50 μL NCs or h^3^-FNCs (50 μg/mL) were dripped onto the wounds of mice in Group 3 or Group 4, respectively, and every 3 days thereafter. PBS (50 μL) was added to the wound of the mice in Group 1 and Group 2 as a control treatment. The body weight and the wounds of the mice were recorded daily. The lesions were photographed during the treatment to observe the healing condition. ROS staining was performed 2 h after the first administration. Some mice were executed on day 2 and the skin tissues were collected and homogenized for bacterial colony analysis. At the end of treatment, the lesioned skin samples were harvested for histopathological H&E, Masson and IHC staining analysis. For biosafety assessment, blood from each mouse was collected for serum biochemical tests and all the mice were dissected to take major organs (heart, liver, spleen, lungs, kidneys) for H&E staining.

**S1.8 Statistical analysis**

The statistical analysis was conducted in triplicate unless otherwise mentioned. The data were reported as mean values ± standard deviation (SD). GraphPad Prism 8, Origin 8.0 and Image J version 1.53c were used for data processing and statistical analysis. Statistical significance was analyzed via one-way ANOVA with Tukey’s multiple comparisons test.

**S2 Supplementary Figures**

**Fig. S1** TEM images of h^3^-FNCs

**Fig. S2** TEM images of ZIF-8

**Fig. S3** The enlarged AC HAADF-STEM image shows the Fe atoms (red circles) dispersed in the NC substrate

**Fig. S4** Fe K-edge EXAFS fitting results of h^3^-FNCs in k space without a phase-shift correction

**Fig. S5** Cumulative pore volumes (**a**) and pore size distribution profiles (**b**) of h^3^-FNCs

**

**

**Fig. S6** FT-IR spectra of the catalysts

**

**

**Fig. S7** N 1s spectra of h^3^-FNCs

**Fig. S8 Catalytic performance of catalysts.** (**a**) Oxidase-like catalytic performance of different catalysts (1 ppm) tested with varied concentrations of TMB. (**b**) Oxidase-like catalytic performance of different catalysts of varied concentrations in TMB (2 mM). (**c**) Time-dependent absorbance changes of TMB (2 mM) in the presence of different concentrations of h^3^-FNCs in deionized water. (**d**) Absorption intensity of TMB (2 mM) in the presence of different catalysts (0.1 ppm) for 30 minutes. (**e**) Lineweaver‒Burk plots for catalysts at varied concentrations of TMB (0-1 mM). (**f**) Oxidase-like activity of h3-FNCs (2 ppm) in water and different pH of HAc-NaAc buffer containing TMB (0.4 mM)


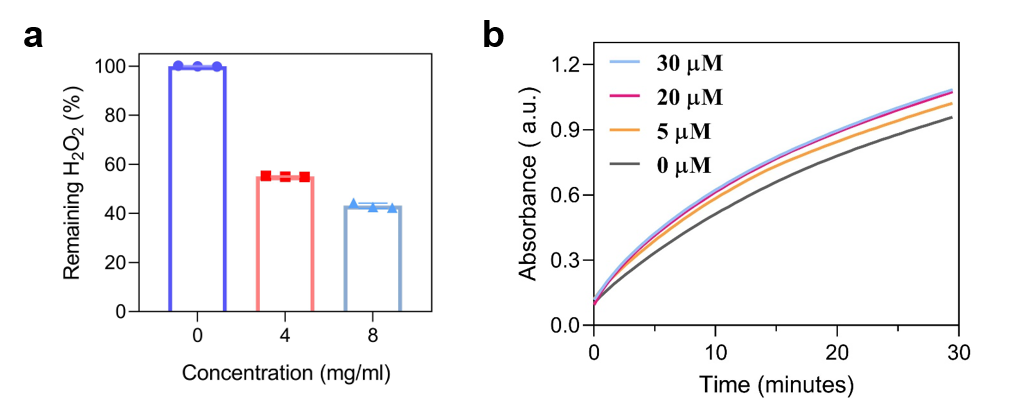


**Fig. S9** (**a**) Proportion of H_2_O_2_ remaining of solutions containing different concentrations of h^3^-FNCs tested by Catalase Assay Kit. (**b**) Oxidase-like activity of h^3^-FNCs (2 ppm) in water and different pH of HAc-NaAc buffer containing TMB (0.4 mM)

**
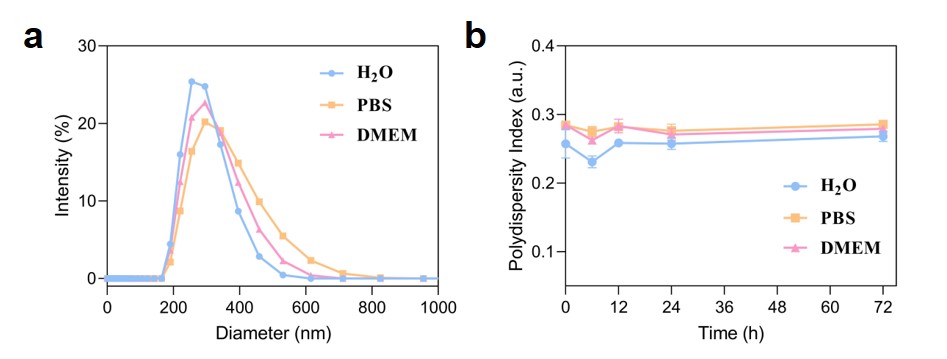
**

**Fig. S10** (**a**) The hydrodynamic diameter of h^3^-FNCs in DI water, PBS (10% FBS) and DMEM medium (10% FBS). (**b**) The stability of h^3^-FNCs in different media (concentration of h^3^-FNCs: 20 μg/mL)

**Fig. S11** Time-dependent absorbance changes of GSH depletion by different concentrations of h^3^-FNCs using DTNB as the indicator


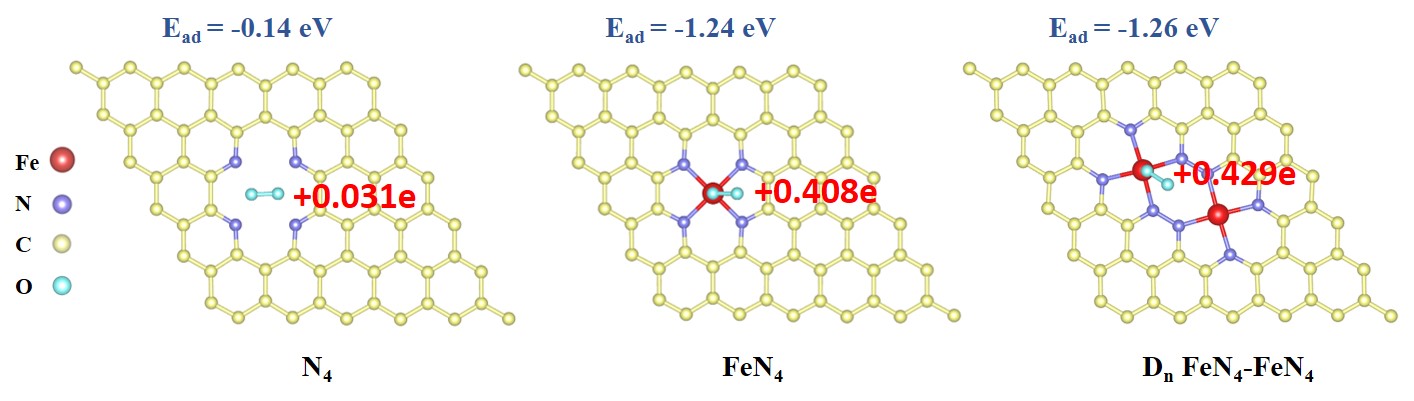


**Fig. S12** Optimal O_2_ adsorption configurations and the E_ad_ of N_4_, Fe–N_4_ and D_n_ FeN_4_–FeN_4_. The red numbers represent the electrons transferred to oxygen

**Fig. S13** SEM images of *S. aureus* and *P. aeruginosa* after different treatments

**
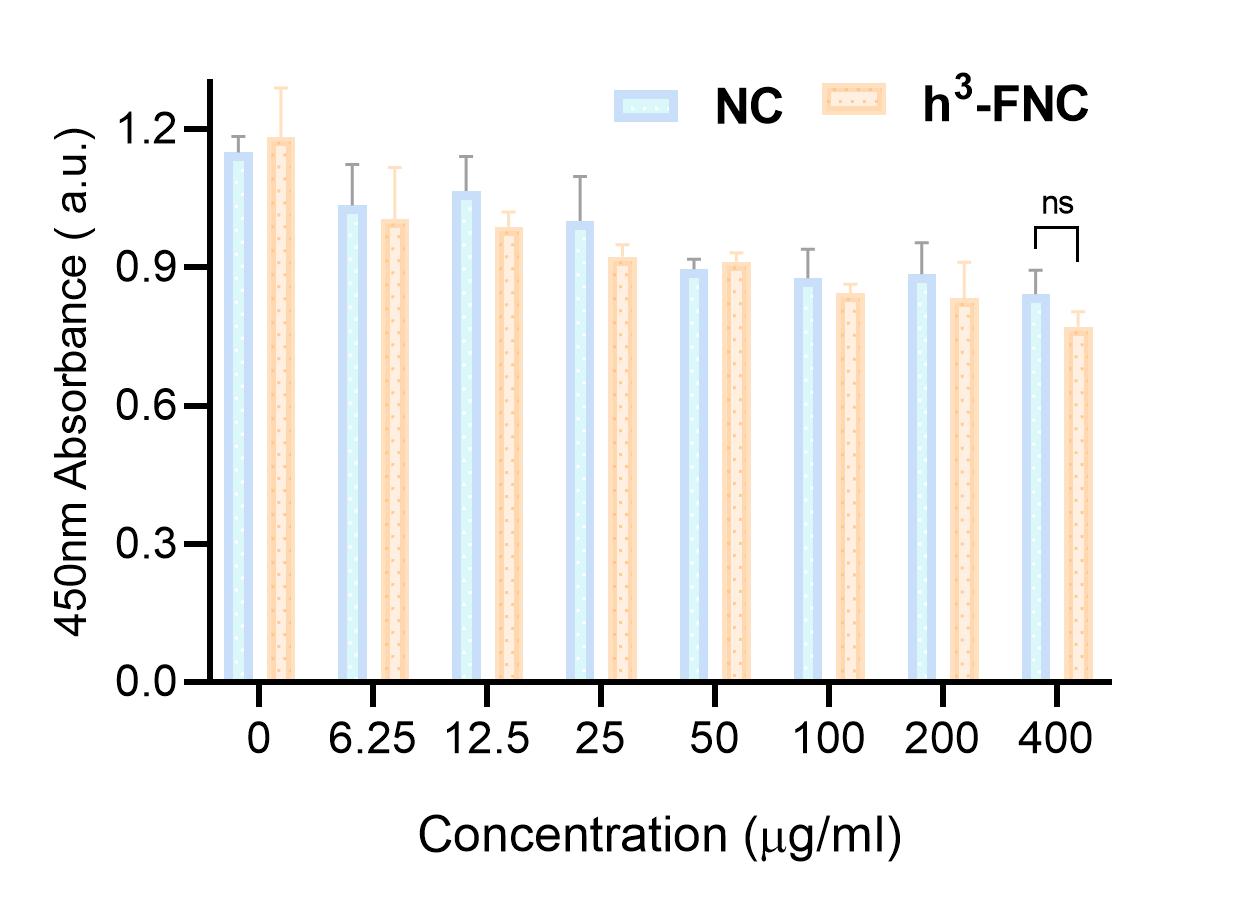
**

**Fig. S14** Absorbance of the HSF cells treated with different concentrations of NCs and h^3^-FNCs. Data are presented as mean values ± SD (n = 3)

**
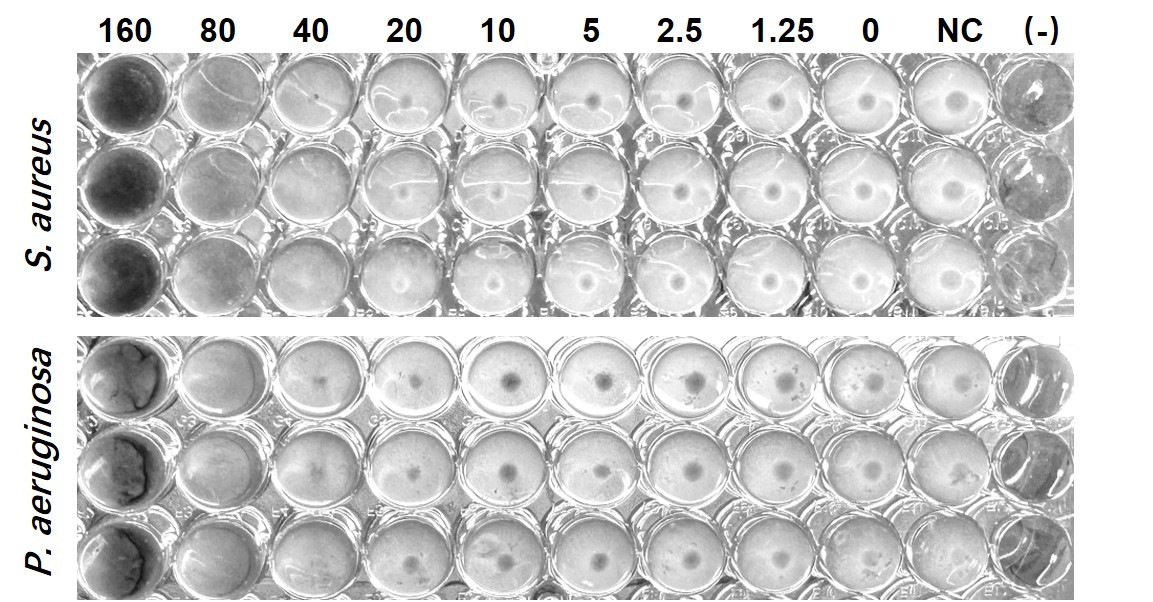
**

**Fig.S15** Minimal inhibition concentrations of h^3^-FNC (μg/mL), and NC (160 μg/mL) against *S. aureus* and *P. aeruginosa*. (-): LB broth without bacteria as the negative control


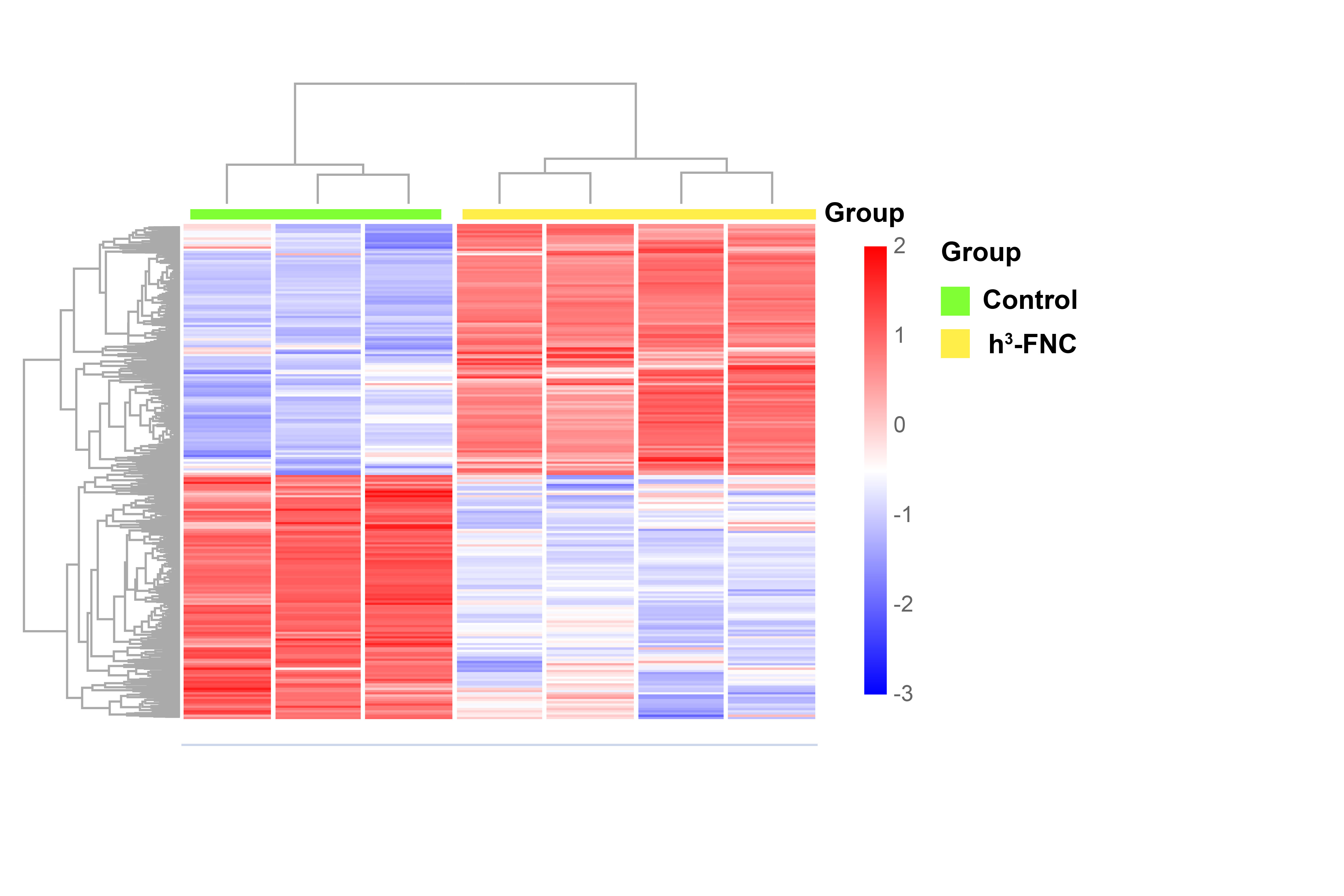


**Fig. S16** Hierarchical clustered heatmap of gene expression profile for control and h^3^-FNC groups


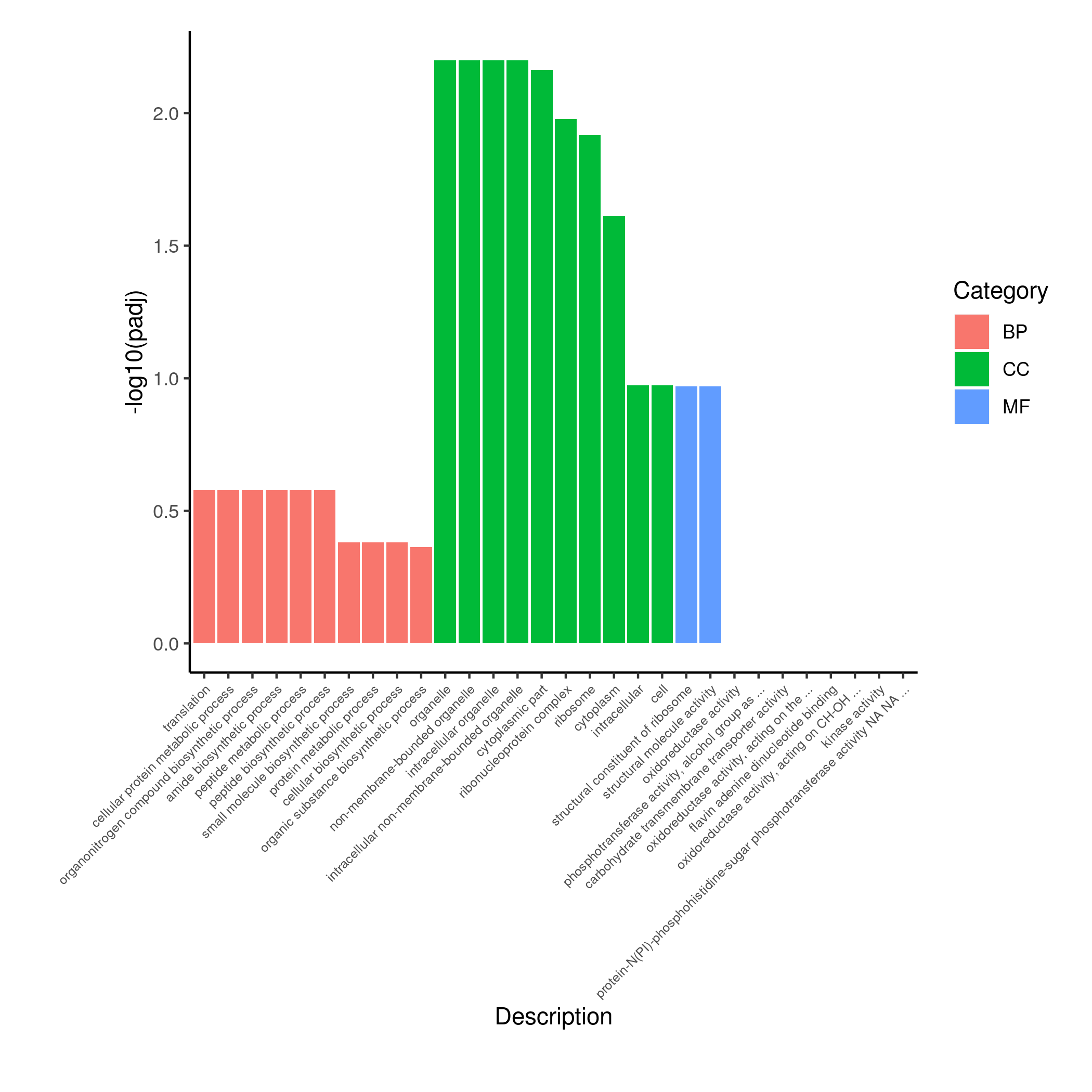


**Fig. S17** Gene function classification for GO analysis

**
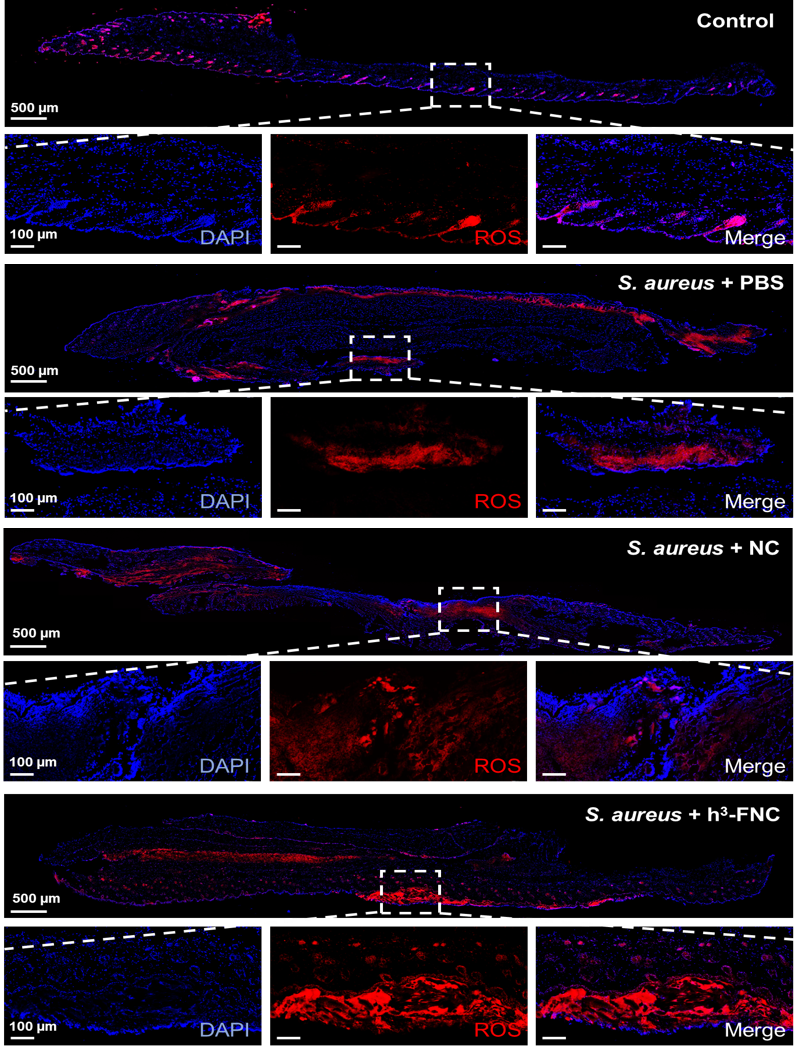
**

**Fig. S18** ROS immunoﬂuorescence staining of skin wound tissues in diﬀerent groups


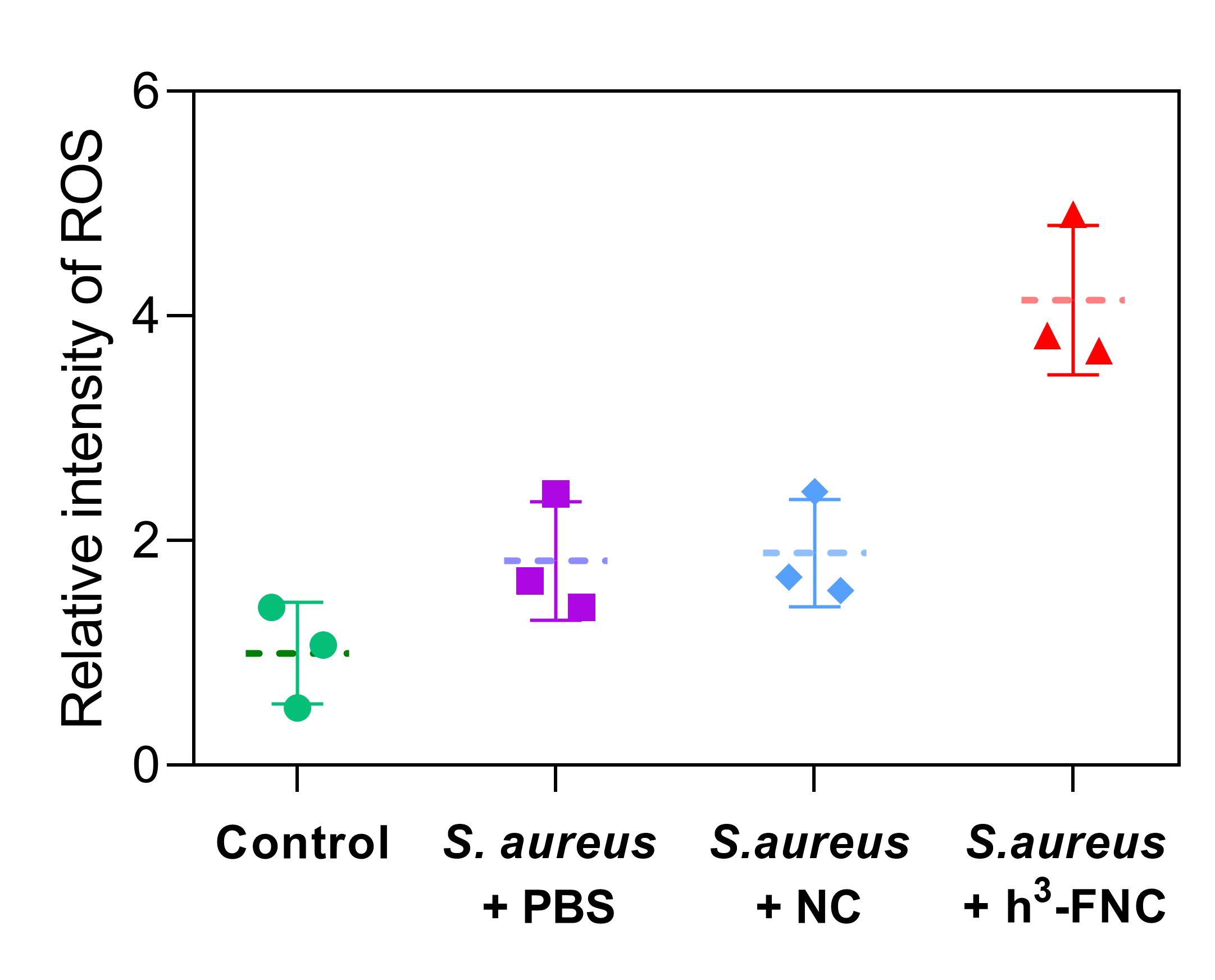


**Fig. S19** Quantitative analysis of ROS immunoﬂuorescence staining of wound tissues

**Fig. S20** Numbers of surviving bacteria in the wound tissues of different groups in 2 days of treatment

**
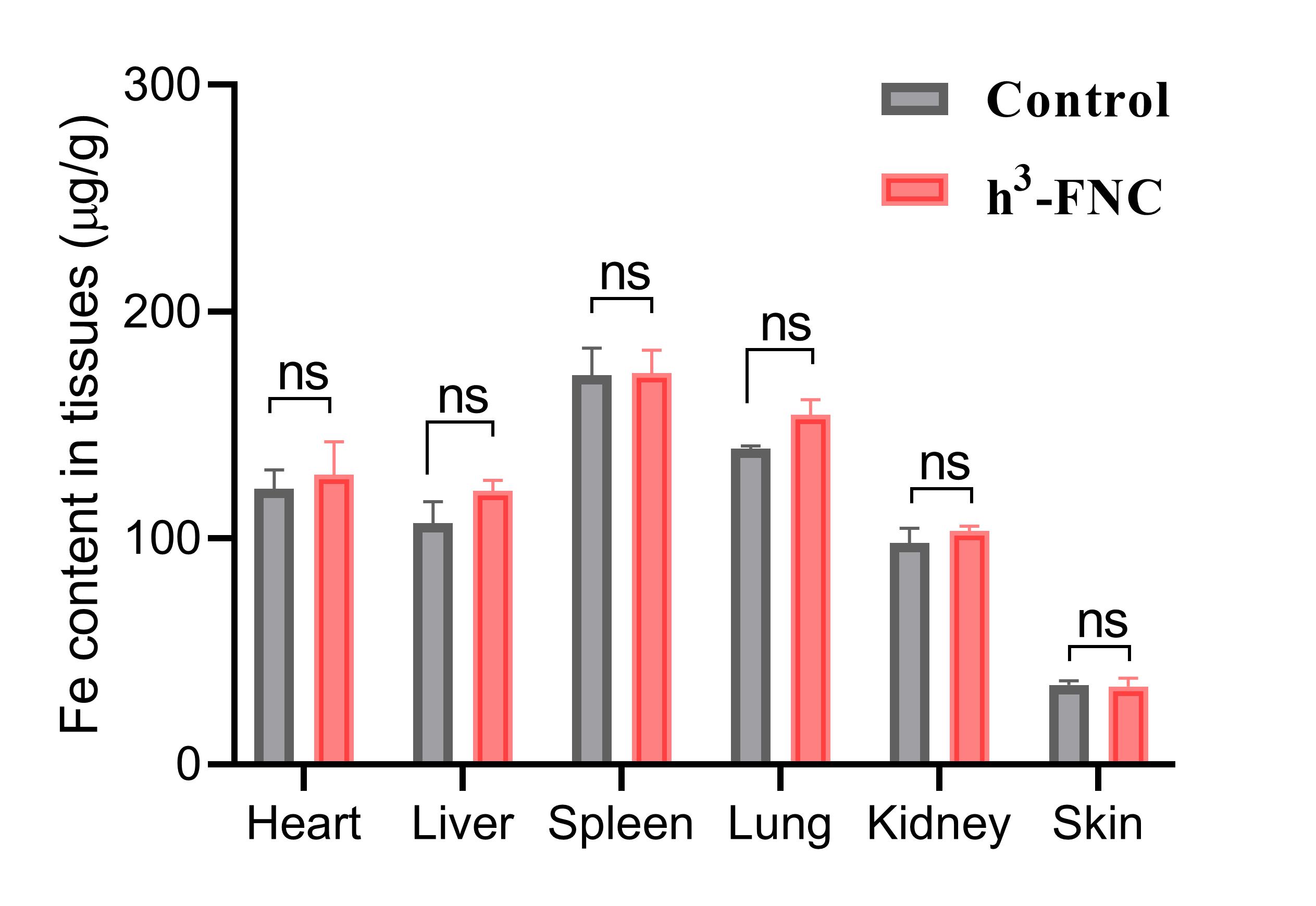
**

**Fig. S21** Fe content in heart, liver, spleen, lung, kidney and skin tissue at the wound site of control and h^3^-FNC group after 4 days of initial administration tested by ICP-OES (n=4)

**
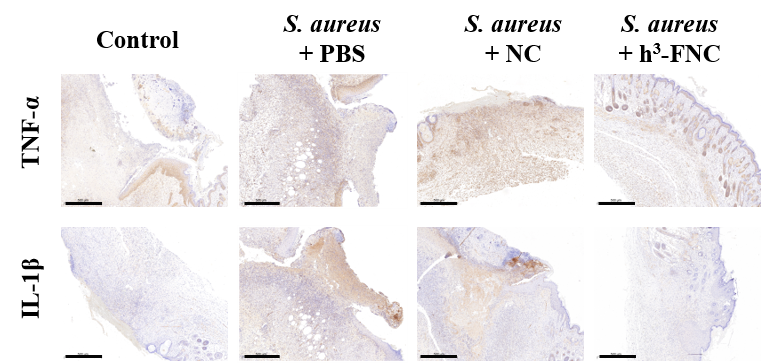
**

**Fig. S22** Representative images of IHC staining for TNF-α (i) and (j) IL-1β expression in the wound tissues of different groups. Scale bar: 500 μm

**Fig. S23** The blood test panel of mice in 10-day treatment

**Fig. S24** H&E staining images of major organs after different treatments. Scale bars: 100 μm

**S3 Supplementary Tables**

**Table S1** The mass content of single atom catalysts in the reported biomedical studies.

| **Catalysts** | **Mass content** | **Applications** | **References** |
| --- | --- | --- | --- |
| PSAF NCs | **Fe: 1.54 wt %** | Tumor therapy | ACS Nano, 2019, 13, 2643 |
| Cu-HNCS | **Cu: 0.18 wt%** | Tumor therapy | Adv. Mater., 2020, 2002246 |
| FeN_5_ SA/CNF | **Fe: 1.2 wt%** | Antibacterial therapy | Sci. Adv., 2019, 5: eaav5490 |
| Zn-SAzyme | **Zn: 3.12 wt%** | Wound disinfection | Angew. Chem., Int. Ed., 2019, 58, 4911 |
| FeN_3_P-SAzyme | **Fe: 0.84 at%** | Tumor therapy | Nat. Catal., 2021, 4, 407–417 |
| Co/PMCS | **Co: 2.02 wt %** | Sepsis management | Angew. Chem., Int. Ed., 2020, 59, 5108–5115 |
| Co-SAs@NC | **Co: 0.506 wt%** | Tumor therapy | Angew. Chem. Int. Ed., 2022, 61, e202204502 |
| Rh/VN_4_ | **Ru: 0.19 wt%** | Brain trauma | Nat. Commun., 2022, 13, 4744 |
| Mn/PSAE | **Mn: /** | Tumor therapy | Angew. Chem., Int. Ed., 2021, 60, 9480–9488 |
| Pb-SACs | **Pb: 0.18 wt%** | Tumor therapy | Angew. Chem., Int. Ed., 2021, 60, 12971–12979 |
| Pt_TS_-SAzyme | **Pt: 0.07 wt%** | Antibacterial therapy | J. Am. Chem. Soc., 2021, 143, 44, 18643 |
| Fe-SANzyme | **Fe: 0.45 wt%** | Retinal vasculopathies | Adv. Mater. 2022, 34, 2205324 |
| Co/TiO_2_ SAzyme | **Co: 1.6 wt%** | Tumor therapy | Adv. Funct. Mater., 2022, 32, 2200331 |
| FeN_5_ SAzyme | **Fe: 0.29 at%** | Tumor therapy | Adv. Mater., 2022, 34, 2107088 |
| Ir-N_5_ SAzyme | **Ir: /** | Tumor therapy | Adv. Mater., 2023, 35, 2208512 |
| BL@B-SA_50_ | **Fe: 1.31 wt%** | Anti-inflammation | Nat. Nanotechnol., 2023, 18, 617–627 |
| CuCH-NCs | **Cu:** **/** | Tumor therapy | Adv. Mater., 2023, 35, 2210201 |
| Ir-SAC | **Ir: 0.11 wt%** | Tumor therapy | Adv. Mater., 2023, 35, 2210037 |
| Mn/QD SAC | **Mn: 0.30 wt%** | Anti-inflammation | Nat. Commun., 2023, 14, 197 |
| Cu_SA_C_6_N_6_ | **Cu: 2.36 wt%** | Biosensing | Nat. Commun., 2023, 14, 2780 |

PSAF NCs: PEGylated single-atom Fe-containing nanocatalysts; Cu-HNCS: Hollow N-doped carbon sphere doped with a single-atom copper species; FeN_5_ SA/CNF: single-atom nanozymes with carbon nanoframe-confined FeN_5_ active centers; Pt_TS_-SAzyme: thermally stable Pt single-atom nanozyme; BL@B-SA_50_: Artificial-enzymes-armed *Bifidobacterium longum* probiotics; QD: Quantum dots.

**Table S2** EXAFS fitting parameters at the Fe K-edge (*Ѕ*_0_^2^=0.72)

| Sample | Path | C.N. | R (Å) | σ^2^×10^3^ (Å^2^) | ΔE (eV) | R factor |
| --- | --- | --- | --- | --- | --- | --- |
| Fe | Fe–N | 4.0* | 2.03±0.03 | 4.8±3.6 | -1.7±4.8 | 0.019 |
|  | Fe–O | 1.0* | 1.85±0.09 |  |  |  |

*C.N*: coordination numbers; *R*: bond distance; *σ*^2^: Debye-Waller factors; Δ*E*: the inner potential correction. *R* factor: goodness of fit. * fitting with fixed parameter

**Table S3** Fitted components of the 57Fe Mӧssbauer spectra obtained at room temperature of h^3^-FNCs

| Catalyst | Component | Rel. Area (%) | δ (mm/s) | QS (mm/s) | W+ (mm/s) |
| --- | --- | --- | --- | --- | --- |
| h^3^-FNCs | D1 | 89.9 | 0.306 | 0.84 | 0.368 |
|  | D2 | 10.1 | 0.47 | 1.65 | 0.209798* |

δ: Isomer shifts; QS: Quadrupole Splitting; W+: Half width at half-maximum (HWHM).

**Table S4** Comparison of the kinetic constants of the catalysts

| Catalysts | K_m_  (mM) | ν_max_  (µM s^-1^) | K_cat_  (10^-1^ s^-1^) | K_cat_/K_m_  (M^-1^ s^-1^) |
| --- | --- | --- | --- | --- |
| h^3^-FNC | 0.46 | 0.34 | 3.03 | 659 |
| m-FNC | 0.31 | 0.07 | 0.92 | 297 |
| l-FNC | 0.17 | 0.01 | 0.67 | 394 |
| 30% Pt/C | 0.14 | 2.2*10^-3^ | 1.43*10^-2^ | 10.2 |

K_m_ is the Michaelis constant, ν_max_ is the maximal reaction velocity and K_cat_ is the catalytic constant, where K_cat_ = ν_max_/[M], [M] is the molar concentration of metal in nanozymes.

**Table S5** Comparison of mass activity of h^3^-FNCs with other oxidase-like catalysts

| Catalysts | Mass activity  (M s^-1^g^-1^ mL) | References |
| --- | --- | --- |
| h^3^-FNC | 0.15 | This work |
| m-FNC | 0.04 | This work |
| l-FNC | 7.21×10^-3^ | This work |
| Commercial Pt/C | 2.22×10^-3^ | / |
| Ag@MnO_2_ | 1.93×10^-4^ | [S7] |
| Cu-HNCS | 0.01 | [S8] |
| Co-SAs@NC | 5.73×10^-3^ | [S9] |
| Co-SAC | 0.06 | [S10] |
| NiSe_2_ | 8.24×10^-3^ | [S11] |
| QAU-Z1 | 5.10×10^-3^ | [S12] |
| FeN_5_ SA/CNF | 0.14 | [S13] |
| Fe_2_NC@Se | 1.76×10^-3^ | [S14] |
| OPA-CsPbBr_3_ NCs | 4.58×10^-5^ | [S15] |
| Co–N_3_(C) | 0.02 | [S16] |
| CoMnO_3_ NFs | 0.02 | [S17] |

**Table S6** Leaching content of Fe in h^3^-FNCs immersing in different solutions by ICP-MS

| The pH of PBS | Mass of sample (g) | Fe concentration in solution (mg/L) | Fe content  (mg/kg) |
| --- | --- | --- | --- |
| 5 | 1.02 | 0 | 0.00 |
| 7.4 | 1.13 | 0 | 0.00 |
| 8 | 1.00 | 0 | 0.00 |

**Table S7** Results for electron transfer during *OOH/*O/*OH adsorption respectively on Fe–N_4_ and N_4_ obtained by Bader charge analysis

| Configuration | Site | ΔQ (e) | | |
| --- | --- | --- | --- | --- |
|  |  | **X**–**OOH** | **X**–**O** | **X**–**OH** |
| Fe–N_4_ | Fe | -1.528 | -1.601 | -1.518 |
|  | Corresponding intermediate  (*OOH/*O/*OH) | 0.526 | 0.705 | 0.488 |
| N_4_ |  | / | / | / |
|  | Corresponding intermediate  (*OOH/*O/*OH) | 0.035 | 0.325 | 0.089 |

ΔQ: charge transfer to the site. Negative ΔQ means charge transfer from Fe to other molecules. X: Fe–N_4_ or –N_4_ in the reaction process. : the vacant site in the middle of N_4_ structure.

**Table S8** Overpotential calculation result of N_4_, Fe–N_4_, and D_n_ FeN_4_–FeN_4_ site models by DFT (U = 1.23 V, T = 298 K and P = 1 bar)

| Configuration | Overpotential (V) |
| --- | --- |
| N_4_ | 0.84 |
| Fe–N_4_ | 0.66 |
| D_n_ FeN_4_–FeN_4_ | 0.46 |

**Supplementary References**

1. J. K. Li, L. Jiao, E. Wegener, L. L. Richard, E. S. Liu et al., Evolution pathway from iron compounds to Fe_1_(II)-N_4_ sites through gas-phase iron during pyrolysis. J. Am. Chem. Soc. **142**, 1417-1423 (2020). <https://doi.org/10.1021/jacs.9b11197>
2. M. L. Aubrey, B. M. Wiers, S. C. Andrews, T. Sakurai, S. E. Reyes-Lillo et al., Electron delocalization and charge mobility as a function of reduction in a metal-organic framework. Nat. Mater. **17**, 625 (2018). <https://doi.org/10.1038/s41563-018-0098-1>
3. M. D. Segall, P. J. D. Lindan, M. J. Probert, C. J. Pickard, P. J. Hasnip et al., First-principles simulation: Ideas, illustrations and the castep code. J. Phys-Condens. Mat. **14**, 2717-2744 (2002). https://doi.org/Pii S0953-8984(02)32831-5
4. P. E. Blochl. Projector augmented-wave method. Phys. Rev. B. **50**, 17953-17979 (1994). [https://doi.org/DOI 10.1103/PhysRevB.50.17953](https://doi.org/DOI%2010.1103/PhysRevB.50.17953)
5. J. P. Perdew, K. Burke, M. Ernzerhof. Generalized gradient approximation made simple. Phys. Rev. Lett. **77**, 3865-3868 (1996). [https://doi.org/DOI 10.1103/PhysRevLett.77.3865](https://doi.org/DOI%2010.1103/PhysRevLett.77.3865)
6. S. Grimme, S. Ehrlich, L. Goerigk. Effect of the damping function in dispersion corrected density functional theory. J. Comput. Chem. **32**, 1456-1465 (2011). <https://doi.org/10.1002/jcc.21759>
7. L. Tian, Z. Huang, W. Na, Y. Liu, S. Wang et al., Heterojunction MnO_2_-nanosheet-decorated Ag nanowires with enhanced oxidase-like activity for the sensitive dual-mode detection of glutathione. Nanoscale **14**, 15340-15347 (2022). <https://doi.org/10.1039/D2NR04294K>
8. X. Y. Lu, S. S. Gao, H. Lin, L. D. Yu, Y. H. Han et al., Bioinspired copper single-atom catalysts for tumor parallel catalytic therapy. Adv. Mater. **32**, 2002246 (2020). https://doi.org/ARTN 2002246
9. S. Cai, J. Liu, J. Ding, Z. Fu, H. Li et al., Tumor-microenvironment-responsive cascade reactions by a cobalt-single-atom nanozyme for synergistic nanocatalytic chemotherapy. Angew. Chem. Int. Ed. **61**, e202204502 (2022). <https://doi.org/10.1002/anie.202204502>
10. X. L. Liu, Q. Liu, X. J. He, G. J. Yang, X. Chen et al., NIR-II-enhanced single-atom-nanozyme for sustainable accelerating bacteria-infected wound healing. Appl. Surf. Sci. **612**, 155866 (2023). [https://doi.org/ARTN 155866](https://doi.org/ARTN%20155866)
11. L. P. Luo, C. Y. Xi, J. C. Zhuo, G. Q. Liu, S. L. Yang et al., A portable dual-mode colorimetric platform for sensitive detection of Hg^2+^ based on NiSe_2_ with Hg^2+^-activated oxidase-like activity. Biosens. Bioelectron. **215**, 114519 (2022). [https://doi.org/ARTN 114519](https://doi.org/ARTN%20114519)
12. D. Q. Zhu, M. L. Zhang, L. Pu, P. P. Gai, F. Li. Nitrogen-enriched conjugated polymer enabled metal-free carbon nanozymes with efficient oxidase-like activity. Small **18**, 2104993 (2022). [https://doi.org/ARTN 2104993](https://doi.org/ARTN%202104993)
13. L. Huang, J. X. Chen, L. F. Gan, J. Wang, S. J. Dong. Single-atom nanozymes. Sci. Adv. **5**, eaav5490 (2019). [https://doi.org/ARTN eaav5490](https://doi.org/ARTN%20eaav5490)
14. R. Z. Tian, H. Y. Ma, W. Ye, Y. J. Li, S. P. Wang et al., Se-containing MOF coated dual-Fe-atom nanozymes with multi-enzyme cascade activities protect against cerebral ischemic reperfusion injury. Adv. Funct. Mater. **32**, 2204025 (2022). [https://doi.org/ARTN 2204025](https://doi.org/ARTN%202204025)
15. Q. Y. Ye, E. X. Yuan, J. Shen, M. L. Ye, Q. Xu et al., Amphiphilic polymer capped perovskite compositing with nano Zr-MOF for nanozyme-involved biomimetic cascade catalysis. Adv. Sci. **10**, 2304149 (2023). [https://doi.org/ARTN 2304149](https://doi.org/ARTN%202304149)
16. Z. Li, F. N. Liu, C. X. Chen, Y. Y. Jiang, P. J. Ni et al., Regulating the N coordination environment of Co single-atom nanozymes for highly efficient oxidase mimics. Nano Lett. **23**, 1505-1513 (2023). <https://doi.org/10.1021/acs.nanolett.2c04944>
17. Z. J. Peng, Y. C. Xiong, Z. W. Liao, M. Zeng, J. L. Zhong et al., Rapid colorimetric detection of H_2_O_2_ in living cells and its upstream series of molecules based on oxidase-like activity of CoMnO_3_ nanofibers. Sensor Actuat. B-Chem. **382**, 133540 (2023). https://doi.org/ARTN 133540
